# Novel Azocoumarin Derivatives—Synthesis and Characterization

**DOI:** 10.3390/ijms23105767

**Published:** 2022-05-21

**Authors:** Katarzyna Piechowska, Angelika Baranowska-Łączkowska, Krzysztof Z. Łączkowski, Jolanta Konieczkowska, Mariola Siwy, Marharyta Vasylieva, Paweł Gnida, Paweł Nitschke, Ewa Schab-Balcerzak

**Affiliations:** 1 Department of Chemical Technology and Pharmaceuticals, Faculty of Pharmacy, Collegium Medicum, Nicolaus Copernicus University, Jurasza 2, 85-089 Bydgoszcz, Poland; kpiechowska@cm.umk.pl; 2 Institute of Physics, Kazimierz Wielki University, Powstańców Wielkopolskich 2, 85-090 Bydgoszcz, Poland; anxela@ukw.edu.pl; 3 Centre of Polymer and Carbon Materials, Polish Academy of Sciences, 34 M. Curie-Skłodowska Str., 41-819 Zabrze, Poland; jkonieczkowska@cmpw-pan.edu.pl (J.K.); msiwy@cmpw-pan.edu.pl (M.S.); marharyta.vasylieva@gmail.com (M.V.); pgnida@cmpw-pan.edu.pl (P.G.); pawel.nitschke@gmail.com (P.N.)

**Keywords:** azocoumarine, photoisomerization reaction, *cis*-*trans* thermal relaxation, photovoltaic cells

## Abstract

The paper presents synthesis and characterization of nine new thiazolyl-(phenyldiazenyl)-2*H*-chromen-2-one dyes. The impact of substituent structure in thiazole ring in the synthesized azocoumarin derivatives on electrochemical properties, photoisomerization process and photovoltaic response was examined. The dyes were electrochemically active and undergo reduction and oxidation processes. They showed low electrochemically estimated energy band gap in the range of 1.71–2.13 eV. Photoisomerization process of the synthesized molecules was studied in various solvents such as ethanol, chloroform and *N*,*N*-dimethylformamide (DMF) upon the UV illumination. It was found that novel azodyes showed reversible *trans*-*cis*-*trans* isomerization and exhibited long thermal back to the trans form, that was even 7 days in DMF. Selected azocoumarin were molecularly dispersed in polystyrene for preparation of guest-host azopolymer systems to study the *cis*-*trans* thermal isomerization of obtained dyes in solid state. The photovoltaic activity of the azochromophores was tested in bulk-heterojunction solar cells. They acting as weak donors in device with structure ITO/PEDOT:PSS/dye:PC70BM/Al. No photovoltaic response of cells with azocoumarin derivatives bearing 4-fluorobenzene, 3,4-dichlorobenzene, or 4-(1-adamantyl) unit was found. Additionally, dye which showed the best activity was examined in three-component solar cells ITO/PEDOT:PSS/PTB7:PC70BM:dye/PFN/Al.

## 1. Introduction

**Scheme 1:**
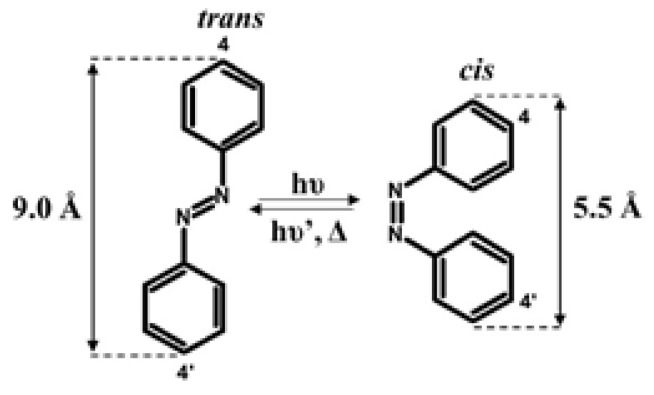
Photoisomerization of azobenzene.

Azo dyes are a class of compounds consisting of azo moiety (–N=N–), which is able to alter its geometry through thermal or photochemical *trans*–*cis* (E⇌Z) conversion (Scheme 1). 

Azo-materials show excellent optical switching properties, making them suitable for application in such areas as optical data storage, liquid crystal displays, or holographic surface relief gratings [1,2,3,4,5,6]. Moreover, the introduction of the azo moieties may give rise to a strong light-absorbing chromophore [7], what is advantageous, considering application in photovoltaics (PV). The activity of the azo dyes in bulk heterojunction solar cells (BHJ) has been already reported [8,9,10], where devices utilizing such compounds as donor components with fullerene acceptor achieved an efficiency of 3.54%. Much worse performance of BHJ has been reported, when azo dyes have been considered for a non-fullerene acceptor, with poly(3-octylthiophene) donor, where such devices have shown efficiency up to 0.65% [11]. Apart from this, compounds consisting of azo-moiety have been investigated as a sensitizer in dye-sensitized solar cells (DSSC) [12,13] or as a hole transporting material (HTM) in perovskite solar cells (PSC) [14].

Despite, the many literature reports on the photoisomerization of azobenzene compounds, the reversible *trans*-*cis*-*trans* isomerization of azocumarins was reported only in one paper, to the best of our knowledge [15]. The described compounds reached photostationary state after ca. 46 s of UV illumination and exhibited long *cis*-*trans* relaxation time (13 h and more) in chloroform, after turning off the excitation light. The conversion efficiency to the cis form was 44–63%. The isomerization of compounds with azocoumarin structure has not been studied in the solid state. Considering the literature, such type of azocompounds has not been tested also in photovoltaic devices. 

Herein, the synthesis and study of electrochemical behavior, *cis*-*trans* isomerization in different solvents and in solid state, and PV activity of new azocoumarin derivatives series is presented. The investigations are focused on effect of substituents structure presents in thiazole ring in new dyes on UV-vis absorption range, frontier molecular orbital energy, ability for photoisomerization process and acting as donor or acceptor in organic photovoltaic BHJ cells.

## 2. Results and Discussion

### 2.1. Synthesis and Structural Characterization

**Figure 1:**
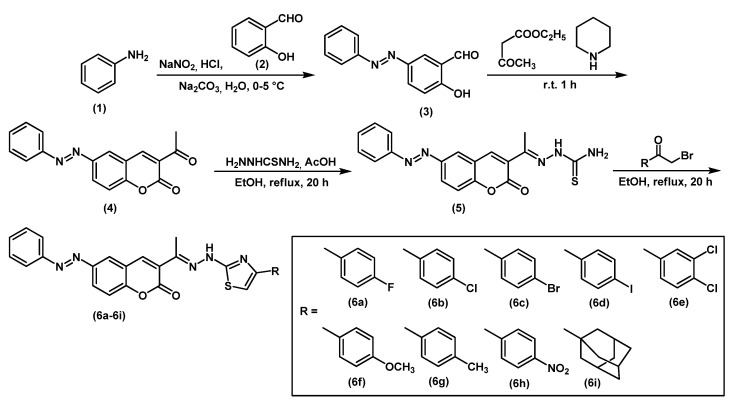
Synthesis route of the target thiazolyl-(phenyldiazenyl)-2H-chromen-2-one dyes (**6a**–**6i**).

The synthesis of designed azocoumarin derivatives involved a three reaction steps. The reaction pathway of new azocompounds is summarized in Figure 1.

In the first step 2-hydroxy-5-(phenyldiazenyl)benzaldehyde (**3**) was prepared by the coupling of a salicylic aldehyde with diazotized aniline in sodium carbonate solution with 34% yield [16]. The ^1^H NMR spectrum confirmed the presence of the CHO and OH groups at 10.38 and 11.51 ppm, respectively. Heating of 2-hydroxy-5-(phenyldiazenyl)benzaldehyde (**3**) with ethyl acetoacetate in absolute ethanol containing a catalytic amount of piperidine led to 3-acetyl-6-(phenyldiazenyl)-2*H*-chromen-2-one (**4**) with a good yield of 65 % [17]. The formation of the desired product was confirmed by the ^1^H NMR spectrum where the disappearance of the CHO and OH signals and the appearance of a singlet at 2.62 ppm derived from the methyl group was observed. Next, 2-(1-(2-oxo-6-(phenyldiazenyl)-2*H*-chromen-3-yl)ethylidene)hydrazinecarbothioamide (5) was synthesized with 65% yield through condensation of 3-acetyl-6-(phenyldiazenyl)-2*H*-chromen-2-one (**4**) with thiosemicarbazide in dry ethyl alcohol containing catalytic amount of glacial acetic acid. In the ^1^H NMR spectrum of hydrazinecarbothioamide **5**, the three signals at 8.46, 8.68 and 10.51 ppm were assigned to the protons of the NH_2_ and NH groups, respectively. Thiazolyl-(phenyldiazenyl)-2*H*-chromen-2-one dyes (**6a**–**6i**) were prepared through Hantzsch cyclization reaction of hydrazinecarbothioamide **5** with a variety of bromoketones in ethanolic solution and under reflux with high yield (45–83%). 

The final products, azocoumarin derivatives **6a**–**6i** were characterized employing spectroscopic methods, ^1^H NMR and ^13^C NMR, and ESI-HRMS analysis. Additionally, for compound **6g** the DEPT-135, COSY, HSQC, HMBC and NOESY spectra was measured. In the ^1^H NMR spectra of compounds **6a**–**6i**, thiazole-5H protons signal were observed at about 6.36–8.37 ppm, broadened hydrazine NH singlet at 11.23–11.63 ppm, and signals at 8.29–8.41 ppm correspond to the CH carbon atoms in the 2H-chromen-2-one ring. The conversion of the substrates to the target products was also confirmed by the ^13^C NMR spectra. In particular, the signals from the C-NH group can be observed at about 170 ppm. Due to the poor solubility of the compounds **6e** and **6h**, their ^13^C NMR spectra could not be measured. Moreover, the ESI-HRMS spectra of the products showed peaks corresponding to their molecular [M+H]^+^ ions.

The solubility of prepared azocoumarin derivatives in distillate water, tetrahydrofuran, chloroform, *N*,*N*-dimethylformamide, and methanol was investigated by dissolving 2 mg of the compound in 1ml of the respective solvent (Table S1). Azo compounds exhibited rather poor solubility in most of used solvents. Azocoumarins were soluble only in DMF after the heat, while in other solvents the studied compounds were insoluble or partially soluble, even when heated to the boiling temperature.

### 2.2. Electrochemistry

The electrochemical behavior of the new azocompounds was investigated by cyclic voltammetry (CV) in a dichloromethane (CH_2_Cl_2_). The obtained electrochemical data are given in Table 1.

**Figure 2:**
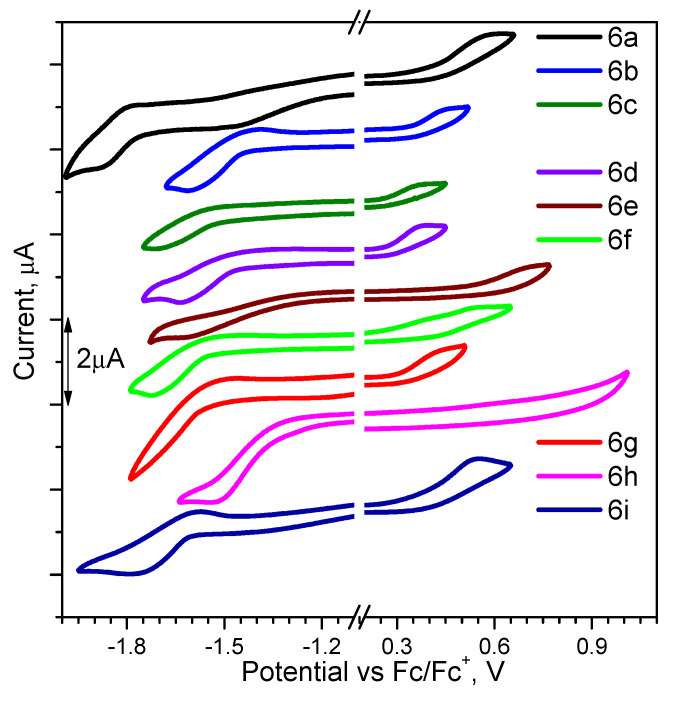
CV curves of **6a**–**6i**. The measurements were performed with a platinum working electrode and referenced against Fc/Fc+ couple, scanning rate 50 mV/s in the electrolyte solution.

In all cases irreversible reduction process was observed. Reduction potentials for compounds bearing 4-chlorobenzen, 4-bromobenzene, 4-iodobenzene, 4-methoxybenzene and 4-methylbenzene (**6b**–**6d**, **6f**, and **6g**) were so closed to each other from −1.53 to −1.42 V (Figure 2). The highest reduction potentials were in case 3,4-dichlorobenzene (**6e**) and 4-nitrobenzene (**6h**) substituted compounds, and the curve of the reduction process was characterized as the lowest current comparing other curves. The lowest oxidation potentials were in case 4-fluorobenzene **6a** (−1.75V) and 4-(1-adamantyl) **6i** (−1.60V) substituted azocoumarin derivatives.

The oxidation process was not observed in case azocoumarin derivative with 4-nitrobenzene group (**6h**) at range 0–1 V. For other cases oxidation processes had irreversible character. The lowest oxidation potential was observed when 4-mehoxybenzene substituent (**6f**) was introduced (0.19 V) and in this case, was the least pronounced oxidation peak. Oxidation potential for other compounds was at a range of 0.25–0.38 V. After several scans any product on the electrode surface was not observed. The energy gap was determined by two methods: using CV curves and using absorbance spectra. Using CV measurements the lowest energy gap was for azo-compounds bearing 4-methoxybenzene (**6f**) and 4-methylbenzene (**6g**) (1.71 eV) groups and the highest in case 4-fluorobenzene (**6a**) substituted azocoumarin (2.13 eV). Optical energy gaps were higher than in the previous case, the lowest was in case of compound with 4-(1-adamantyl) (**6i**) group (2.18 eV) and the highest in case 4-fluorobenzene (**6a**) group (2.40 eV).

### 2.3. Photoisomerization

**Figure 3:**
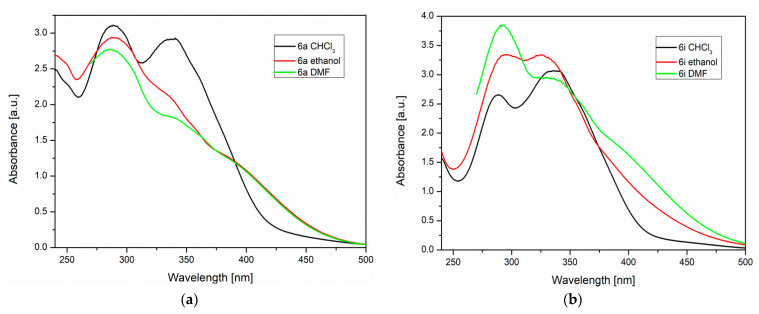
UV spectra in chloroform, ethanol and DMF for (**a**) **6a**, (**b**) **6i**.

The reversible *trans*-*cis*-*trans* isomerization of azocoumarin derivatives was examined in three different solvents: chloroform, ethanol, and DMF (c = 10^−5^ mol/L). The absorption spectra of the compounds were solvent-dependent. In chloroform azocompounds exhibiting two separated absorption bands, weak at ca. 340 nm corresponds to N=N linkage and strong band at ca. 290 nm characteristic for n-π* transition of coumarin [18]. While in ethanol and DMF the absorption band corresponds to azo group was not well-formed. For 4-(1-adamantyl) substituted azocoumarin (**6i**) two absorption bands were observed in all used solvents (Table 2, Figure 3). Azocumarines can form a hydrogen bonds with molecules of ethanol and DMF, what can be observed by (i) a new absorption band in the range of 380–480 nm, and (ii) a slight blue shift of the n-π* band in ethanol and DMF relative to CHCl_3_ [18].

**Figure 4:**
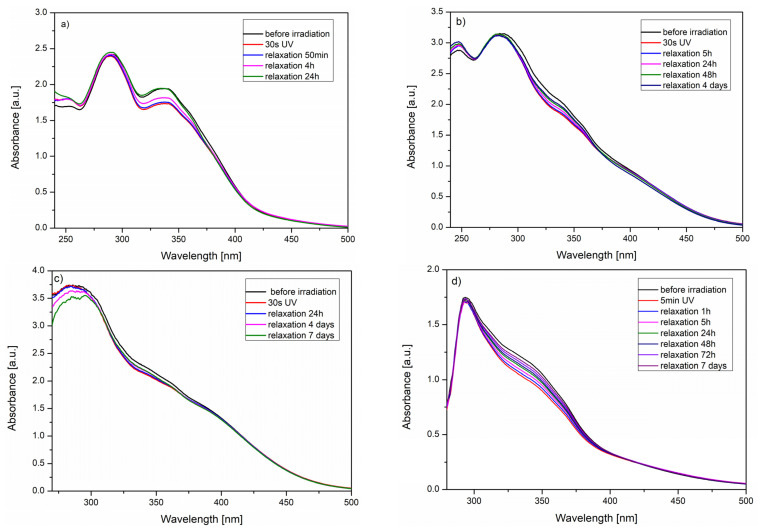
*Cis*-*trans* isomerization for **6b** in (**a**) chloroform, (**b**) ethanol, (**c**) DMF, and (**d**) polystyrene.

*Trans*-*cis* conversion was generated upon the UV diode light irradiation (λ = 365 nm; *P* = 2.9 W) by 30 s. Confirmation of the isomerization process was the reduction in intensity band at ca. 340 nm in comparison to the state before exposure; the presence of isosbestic points; and increase of the band intensity after turning off the excitation light (Table 3, Figure 4 and Figure S1). The *cis*-isomer content was calculated according to Equation (1) directly after 30 s of UV light irradiation (Table 3).

Analyzing the date collected in Table 3, a significant impact of the solvent on isomerization was observed. The fastest *cis*-*trans* reaction was noticed for chloroform, where the full conversion to the trans form took place within maximum 29 h (except for 3,4-dichlorbenze (**6e**) and 4-methoxybenzene (**6f**) substituted compounds). For other solvents the full *cis*-*trans* reaction back was not observed within 4 days for ethanol, and 7 days in DMF. Considering the *cis*-isomer content after UV irradiation, the higher concentration of *cis*-form was noticed for chloroform, and the lowest for DMF. Based on the *cis*-*trans* isomerization times, contents of *cis*-isomer after UV irradiation dates can be concluded, that the whole process of reversible *trans*-*cis*-*trans* isomerization was a significant faster in chloroform, than ethanol and DMF. It can be result of the formation of hydrogen bonds between the molecules of the solvent and azocoumarin compounds which inhibits the isomerization. These non-covalent interactions have a significant effect on the molecule of azocompound, that is observed for UV-Vis curves. The absorption band characteristic for N=N bonds at ca. 340 nm were not observed in ethanol and DMF solvents (cf. Figure 3). The content of *cis*-isomer in chloroform was in the range of 7–22%. Compare to literature results concern azocoumarin derivatives it is lower isomerization efficiency [15]. The differences result from different compounds structure. In our molecules azo bond is directly connected with bulk coumarin unit and it can hinder the isomerization. Moreover, for isomerization of reported azocoumarins [15] power irradiation was 200 W in our investigations we applied 2.9 W. The photoisomerization process was the least efficient in the case of dye **6h**. However, the impact of substituent in thiazole ring was rather not significantly pronounced probably due to its location in azocoumarin structure, which was located away from the N=N bond.

The cis-trans isomerization was studied in the solid state for guest-host systems with azocoumarins dispersed molecularly in polystyrene. The thin films of polymers contained 5 wt.% of azodye were UV irradiated by 5 min and after the turning off the excitation light the dark relaxation was monitored. It was found that the content of cis-isomer was in the range of 7–22% (Table S2). Similar as in solution, the lowest cis-isomer content was found for **6h**. The full cis-trans reaction back was observed within 7 or 8 days in the case of dyes **6f**, **6h**, **6a** and **6b**. In the case of molecules with 4-bromobenzene (**6b**), 4-iodobenzene (**6d**), 4-methylbenzene (**6g**) and 4-(1-adamantyl) (**6i**) the full cis-trans thermal isomerization was not proceeded within 8 dyes.2.4. Photovoltaic Performance

**Figure 5:**
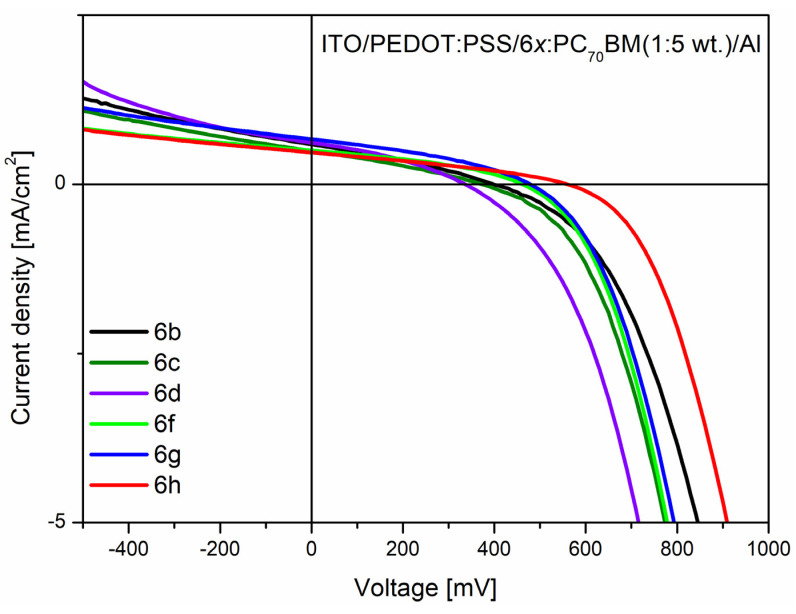
Current density-voltage characteristics of BHJ solar cells with azo-compound donor component based on the registered curves, PV parameters of constructed devices: short circuit current density (J_SC_), open-circuit voltage (V_OC_), fill factor (FF), power conversion efficiency (PCE), series resistance (R_S_), and shunt resistance (R_SH_) were calculated and are gathered in Table 4.

All of the synthesized azocoumarins were tested as component of active layer in in bulk-heterojunction (BHJ) solar cells. The preliminary test revealed that the investigated compounds exhibited activity in devices when acting as donors together with fullerene acceptor in device with structure ITO/PEDOT:PSS/dye:PC70BM/Al. No PV response was observed while they were applied as acceptor component together with standard poly(3-hexylthiophene) donor material. Registered current-density–voltage (J–V) characteristics of the prepared solar cell, consisting of azocoumarin donor (D) component and [6,6]-Phenyl-C71-butyric acid methyl ester (PC_71_BM) acceptor (A) (D:A wt. ratio of 1:5) under illumination are presented in Figure 5. The J–V characteristics of individual solar cells, under illumination and in the dark are shown in Figure S2.

**Figure 6:**
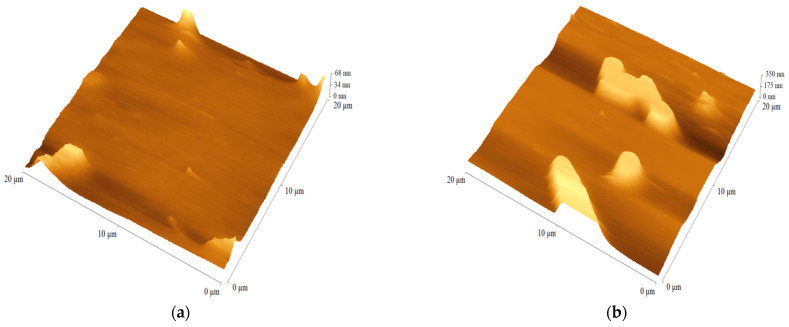
The AFM micrograms of fabricated devices containing **6e** (**a**) or **6h** (**b**) in the active layer.

Analysis of the PV parameters revealed that most of the studied azo-compounds exhibited activity in BHJ devices when acting as a donor. Only 4-fluorobenzene (**6a**), 3,4-dichlorobenzene (**6e**), or 4-(1-adamantyl) (**6i**) substituted molecules did not show any activity in the devices. All of these solar cells have shown similar shunt (R_SH_) and series (R_S_) resistances, what might be a result of poor film-forming properties of the azo-compounds used in these active layers. The devices revealed similar and very low average power conversion efficiencies being in a range of 0.05–0.10%, which is similar to those obtained for other azo-dyes [19,20] or azo-polymers [21] reported in literature. The highest PCE was registered for a PV cell with an active layer consisting of compound with methyl substituent (**6g**) (0.12% max.). Such value is due to one of the highest open-circuit voltages (420 mV). Moreover, the active layer consisting of 4-methylbenzene substituted azocoumarin (**6g**) showed the highest short-circuit current density (0.63 mA/cm^2^), what correlates in a good manner with one of the lowest registered series resistances (6.23 kΩ). The lowest Rs (6.11 kΩ) was detected for BHJ with compound bearing iodine atom (**6d**), presents shown similar J_SC_ (0.61 mA.cm^2^). However, low V_OC_ of 375 mV, decreased the power conversion efficiency to 0.08%. The highest V_OC_ (496 mV) was found for nitro-substituted compound (**6h**), however due to the highest series resistance, the active layer of this particular compound also exhibited the lowest J_SC_ (0.43 mA/cm^2^). Current density values are also, among other things, related to the quality of the thin film. Thus, active layers were examined by atomic force microscopy (AFM) to determine the its thickness and roughness. The roughness is represented by the root-mean-square (RMS) parameter. RMS values were in a relatively wide range from 10 to 55 nm. Active layer containing compounds with iodobenzene (**6d**) and 3,4-dichlorobenzene (**6e**) showed the lowest roughness, 18 and 10 nm, respectively. In contrast, RMS of blends with azcoumarines bearing 4-nitrobenzene (**6h**) and 4-(1-adamantyl) (**6i**) substituents, being 55 and 50 nm, respectively, were the roughest (Figure 6). The device showing the highest efficiency (0.1 %) had a roughness of 40 nm.

The second important element influencing the photovoltaic performance of the devices is the thickness of the active layer. The thickness values of the tested blends ranged from 50 to 65 nm. The lowest thicknesses showed blends containing compounds azocoumarins with 4-iodobenzene (**6d**) 4-methylbenzene (**6g**) or 4-nitrobenzene (**6h**), while the formation of the thickest layers was caused by the presence of compounds beairing 4-bromobenzene (**6c**) or 3,4-dichlorobenzene (**6e**) groups. The discrepancies in thicknesses are not large enough to have a direct significant effect on the photovoltaic performance of the cells tested, more likely the quality of the blends tested was of greater importance.

In the next step of investigations three-component BHJ PV cells were fabricated. Since the best photovoltaic performance was detected for 4-methylbenzene substituted azocoumarin (**6g**), this compound was chosen for to three-component active layer preparation and **6g** was introduced into PTB7:PC70BM system. Two weight ratios of PTB7:PC70BM:**6g** components were tested, namely 4:8:1 and 13:8:1 (Table 1). The fabricated devices exhibited PCE above 2%. A decrease of the **6g** content in active layer from 4:8:1 to 13:8:1 allowed elevating the current density, however not due to a decreased Rs, but as a result of significantly decreased shunt-resistance. Such changes caused a decrease of the fill factor of the device, however, due to elevated current density, device showed higher PCE compare to cell with higher **6g** content.

## 3. Conclusions

The synthesized new azocoumarin derivatives were obtained as solid compounds with high melting temperature being in the range of 195–256 °C. The presence of 3,4-dichlorobenzene (**6e**) and 4-nitrobenzene (6h) lowering (T_m_ = 195 °C) and raising (T_m_ = 256 °C) melting temperature compare to others substituents (T_m_ = 223–239 °C) in thiazole ring, respectively. The effect of substituent structure in thiazole ring in azocoumarin derivatives on reduction and oxidation potentials resulting in E_g_ value was seen. Molecules bearing 4-chlorobenzene (**6b**), 4-bromobenzene (**6c**), 4-iodobenzene (**6d**) and 4-mehoxybenzene (**6f**) characterized by the lowest E_g_ about 1.7 eV. Considering the photoisomerization process it can be concluded that investigated azochromophores create stable *cis*-isomer both in solution and in guest-host systems with PS after UV irradiation and thermal back relaxation proceeds during even more than 7 days in DMF and in PS. It was found that both obtained content of *cis*-isomer and its stability strongly depending on type of solvent contrary to impact of substituent structure which was less pronounced probably due to its location in azocoumarin structure, which was located away from the N=N bond. The conversion to the *cis*-isomer was in the range of 7–22% being the highest in chloroform solution for molecule with 4-mehoxybenzene (**6f**) unit. Azocoumarin derivatives containing the 4-chlorobenzene (**6b**), 3,4-dichlorobenzene (**6e**) and 4-(1-adamantyl) (**6i**) unit did not exhibit the activity in BHJ photovoltaic cells. The application in solar cell 4-methylbenzene substituted azocoumarin (**6g**) resulted in the best PV parameters. Significant increase in PCE exhibited, three-component BHJ device. The lowering of **6g** content in relation to second donor component in active layer enhanced the short circuit current density and open-circuit voltage resulted in increases of device efficiency.

## 4. Materials and Methods

### 4.1. Measurement

^1^H NMR (400 MHz) and ^13^C NMR (100 MHz) spectra were recorded on a Bruker Avance III multinuclear instrument. High resolution mass spectrometry measurements were performed using Synapt G2-Si mass spectrometer (Waters) equipped with quadrupole-Time-of-flight mass analyzer. The mass spectrometer was operated in the positive ion detection mode. The results of the measurements were processed using the MassLynx 4.1 software (Waters) incorporated with the instrument. Analytical TLC was performed using Macherey-Nagel Polygram Sil G/UV254 0.2 mm plates. Differential scanning calorimetry (DSC) was performed with a TA-DSC 2010 apparatus (TA Instruments) under nitrogen using heating/cooling cycles of 20°C/min. Surface images together with thin films’ roughness and thickness have been investigated using an atomic force microscope (AFM) TopoMetrix Explorer device (Industriële Veiling Eindhoven B.V., CA Eindhoven, The Netherlands), working in the contact mode in the air in the constant force regime. The electrochemical cell is comprised of the platinum electrode with a 1 mm diameter of Pt as a working electrode. an Ag|Ag+ electrode as a pseudoreference electrode and a platinum coil as an auxiliary electrode. Measurements were conducted at room temperature at a potential rate of 50 mV/s and were calibrated against a ferrocene/ferrocenium redox couple. Electrochemical measurements were conducted in 1.0 mM concentrations of all compounds for all cyclic voltammetry measurements. Electrochemical studies were undertaken in 0.1 M solutions of Bu_4_NBF_4_. 99% (Sigma Aldrich) in dichloromethane (DCM) CHROMASOLV ^®^. 99.9% (Sigma Aldrich). Research manuscripts reporting large datasets that are deposited in a publicly available database should specify where the data have been deposited and provide the relevant accession numbers. If the accession numbers have not yet been obtained at the time of submission, please state that they will be provided during review. They must be provided prior to publication.

Interventionary studies involving animals or humans, and other studies that require ethical approval, must list the authority that provided approval and the corresponding ethical approval code.

### 4.2. Chemicals

Surfactant (Hellmanex III) was purchased from Hellma Analytics). The solvents - isopropanol and chlorobenzene have been purchased from Avantor Performance Materials and used as received. Poly [[4.8-bis[(2-ethylhexyl)oxy]benzo[1.2-b:4.5-b’]dithiophene-2.6-diyl][3-fluoro-2-[(2-ethylhexyl)carbonyl]thieno[3.4-b]thiophenediyl]] (PTB7) (M214. *M*_w_ = 322,236 g/mol). PEDOT:PSS dispersion in water (M124) and [6.6]-Phenyl-C71-butyric acid methyl ester (PC70BM) have been purchased in Ossila and used as received.

### 4.3. Synthesis and Structural Characterization

All experiments were carried out under an air atmosphere. Reagents were generally best quality commercial-grade products and were used without further purification.

#### 4.3.1. 2-Hydroxy-5-(phenyldiazenyl)benzaldehyde (**3**)

In a 25-mL round bottom flask a mixture containing aniline (1) (5.0 mL. 55.0 mmol) in water (4.0 mL) was cooled in an ice bath and while stirring. Then concentrated hydrochloric acid (6.0 mL) was added dropwise to the reaction mixture within 5 min. After that. 20% sodium nitrite (20.0 mL) in water was added and the mixture was stirred for additional 20 min. Next salicylic aldehyde (2) (5.0 mL. 55.0 mmol) in solution of sodium carbonate (18.0 g) in water (150 mL) was added dropwise to the reaction mixture within 1 h and the reaction mixture was stirred for additional 1.5 h. Solid product was filtered. washed with water (600 mL). dried and was purified on silica gel column chromatography (230–400 mesh) using dichloromethane to afford the desired product. Yield: 4.22 g. 34%. (dichloromethane/methanol. 95:5. R_f_ = 0.74). ^1^H NMR (DMSO-d_6_. 400 MHz). δ (ppm): 7.21 (d. 1H. CH. J = 8.8 Hz); 7.51–7.63 (m. 3H. 3CH); 7.84–7.90 (m. 2H. 2CH); 8.10 (dd. 1H. CH. J_1_ = 2.4 Hz. J_2_ = 8.8 Hz); 8.19 (d. 1H. CH. J = 2.4 Hz); 10.38 (s. 1H. CHO); 11.51 (bs. 1H. OH). ^13^C NMR (DMSO-d_6_. 100 MHz); δ (ppm): 118.87 (C); 122.82 (2C); 123.10 (C); 124.35 (C); 129.87 (2C); 130.14 (C); 131.55 (C); 145.28 (C); 152.37 (C); 163.72 (C); 167.20 (C). 

#### 4.3.2. 3-Acetyl-6-(phenyldiazenyl)-2H-chromen-2-one (**4**)

A mixture of 2-hydroxy-5-(phenyldiazenyl)benzaldehyde (3) (0.80 g. 3.54 mmol) and ethyl acetoacetate (0.46 g. 3.54 mmol) in absolute ethanol (15 mL) containing a catalytic amount of piperidine (0.074 g. 0.87 mmol) was heated under reflux for 2 h. The precipitated solid was collected by filtration and washed with ethyl alcohol to afford the desired product. Yield: 0.67 g. 65%. (dichloromethane. R_f_ = 0.20). ^1^H NMR (DMSO-d_6_. 400 MHz). δ (ppm): 2.62 (s. 3H. CH_3_); 7.60–7.69 (m. 4H. 4CH); 7.90–7.95 (m. 2H. 2CH); 8.23 (dd. 1H. CH. J_1_ = 2.0 Hz. J_2_ = 8.8 Hz); 8.54 (d. 1H. CH. J = 2.4 Hz); 8.83 (s. 1H. CH). ^13^C NMR (DMSO-d_6_. 100 MHz); δ (ppm): 30.42 (C); 117.98 (C); 119.30 (C); 123.12 (2C); 125.80 (C); 126.49 (C); 127.34 (C); 130.02 (2C); 132.38 (C); 147.26 (C); 148.89 (C); 152.27 (C); 156.63 (C); 158.47 (C); 195.48 (C). 

#### 4.3.3. 2-(1-(2-Oxo-6-(phenyldiazenyl)-2H-chromen-3-yl)ethylidene)hydrazinecarbothioamide (**5**)

Thiosemicarbazide (0.16 g. 1.71 mmol) was added to a stirred solution of 3-acetyl-6-(phenyldiazenyl)-2*H*-chromen-2-one (4) (0.50 g. 1.71 mmol) in absolute ethyl alcohol (20 mL) and then (0.15 mL) of acetic acid was added. The reaction mixture was stirred under reflux for 20 h. The precipitated solid was collected by filtration and washed with ethyl alcohol to afford the desired product. Yield: 0.57 g. 65%. (dichloromethane/methanol (95:5). R_f_ = 0.42). ^1^H NMR (DMSO-d_6_. 400 MHz). δ (ppm): 2.29 (s. 3H. CH3); 7.59–7.66 (m. 4H. 4CH); 7.90–7.94 (m. 2H. 2CH); 7.98 (bs. 1H. NH); 8.17 (dd. 1H. CH. J_1_ = 2.4 Hz. J_2_ = 9.2 Hz); 8.30 (d. 1H. CH. J = 2.4 Hz); 8.46 (bs. 1H. NH); 8.68 (s. 1H. CH); 10.51 (bs. 1H. NH). ^13^C NMR (DMSO-d_6_. 100 MHz); δ (ppm): 16.36 (C); 117.74 (C); 120.04 (C); 123.07 (2C); 123.35 (C); 126.87 (C); 126.96 (C); 130.02 (2C); 132.27 (C); 142.24 (C); 145.79 (C); 148.79 (C); 152.28 (C); 155.45 (C); 159.13 (C); 179.12 (C).

#### 4.3.4. 3-(1-(2-(4-(4-Fluorophenyl)thiazol-2-yl)hydrazono)ethyl)-6-(phenyldiazenyl)-2H-chromen-2-one (**6a**) 

Carbothioamide 5 (0.285 g. 0.78 mmol) was added to a stirred solution of 2-bromo-1-(4-chlorophenyl)ethanone (0.182 g. 0.78 mmol) in absolute ethyl alcohol (45 mL). The reaction mixture was stirred under reflux for 20 h. The precipitated solid was collected by filtration and washed with ethyl alcohol to afford the desired product. Yield: 0.22 g. 55%. (dichloromethane/methanol (95:5). R_f_ = 0.74). ^1^H NMR (DMSO-d_6_. 400 MHz). δ (ppm): 2.31 (s. 3H. CH_3_); 7.45 (s. 1H. CH); 7.48 (d. 2H. 2CH. J = 8.8 Hz); 7.59–7.66 (m. 4H. 4CH); 7.88–7.96 (m. 4H. 4CH); 8.15 (dd. 1H. CH. J_1_ = 2.4 Hz. J_2_ = 8.8 Hz); 8.36 (s. 1H. CH); 8.45 (d. 1H. CH. J = 2.0 Hz); 11.44 (bs. 1H. NH). ^13^C NMR (DMSO-d_6_. 100 MHz); δ (ppm): 16.59 (C); 104.71 (C); 115.79 (C); 116.04 (C); 117.78 (C); 120.08 (C); 123.08 (2C); 124.42 (C); 125.81 (C); 127.80 (C); 127.97 (C); 128.05 (C); 130.02 (2C); 132.24 (C); 140.95 (C); 145.16 (C); 148.90 (C); 152.32 (C); 155.39 (C); 159.21 (C); 169.87 (C). ESI-HRMS (m/z) calculated for C_26_H_19_FN_5_O_2_S: 484.1243 [M+H]^+^. Found: 484.1242 [M+H]^+^. T_m_ = 227 °C.

#### 4.3.5. 3-(1-(2-(4-(4-Chlorophenyl)thiazol-2-yl)hydrazono)ethyl)-6-(phenyldiazenyl)-2H-chromen-2-one (**6b**)

Yields: 0.27 g. 66%. (dichloromethane/methanol (95:5). R_f_ = 0.84). ^1^H NMR (DMSO-d_6_. 400 MHz). δ (ppm): 2.33 (s. 3H. CH_3_); 7.49 (s. 1H. CH); 7.62–7.69 (m. 6H. 6CH); 7.86 (d. 2H. 2CH. J = 8.4 Hz); 7.96 (d. 2H. 2CH. J = 8.4 Hz); 8.18 (dd. 1H. CH. J_1_ = 2.1 Hz. J_2_ = 9.1 Hz); 8.39 (s. 1H. CH); 8.48 (d. 1H. CH. J = 2.8 Hz); 11.51 (bs. 1H. NH). ^13^C NMR (DMSO-d_6_. 100 MHz); δ (ppm): 16.59 (C); 105.77 (C); 117.79 (C); 120.06 (C); 123.08 (2C); 124.39 (C); 125.83 (C); 127.74 (2C); 127.76 (C); 129.11 (2C); 130.00 (2C); 132.22 (C); 132.42 (C); 133.99 (C); 140.97 (C); 145.25 (C); 148.88 (C); 152.33 (C); 155.38 (C); 159.17 (C); 169.91 (C). ESI-HRMS (m/z) calculated for C_26_H_19_ClN_5_O_2_S: 500.0948 [M+H]^+^. Found: 500.0941 [M+H]^+^. T_m_ = 223 °C.

#### 4.3.6. 3-(1-(2-(4-(4-Bromophenyl)thiazol-2-yl)hydrazono)ethyl)-6-(phenyldiazenyl)-2H-chromen-2-one (**6c**) 

Yields: 0.19 g. 45%. (dichloromethane/methanol (95:5). R_f_ = 0.76). ^1^H NMR (DMSO-d_6_. 400 MHz). δ (ppm): 2.31 (s. 3H. CH_3_); 7.25 (t. 2H. 2CH. J = 9.2 Hz); 7.36 (s. 1H. CH); 7.59–7.67 (m. 4H. 4CH); 7.88–7.97 (m. 4H. 4CH); 8.15 (dd. 1H. CH. J_1_ = 2.4 Hz. J_2_ = 8.8 Hz); 8.36 (s. 1H. CH); 8.44 (d. 1H. CH. J = 2.4 Hz); 11.42 (bs. 1H. NH). ^13^C NMR (DMSO-d_6_. 100 MHz); δ (ppm): 16.59 (C); 105.86 (C); 117.76 (C); 120.07 (C); 121.01 (C); 123.08 (2C); 124.41 (C); 125.82 (C); 127.76 (C); 128.05 (2C); 130.01 (2C); 132.01 (2C); 132.24 (C); 134.37 (C); 140.98 (C); 145.21 (C); 148.87 (C); 152.31 (C); 155.38 (C); 159.18 (C); 169.91 (C). ESI-HRMS (m/z) calculated for C_26_H_19_BrN_5_O_2_S: 544.0443 [M+H]^+^. Found: 544.0448 [M+H]^+^. T_m_ = 232 °C.

#### 4.3.7. 3-(1-(2-(4-(4-Iodophenyl)thiazol-2-yl)hydrazono)ethyl)-6-(phenyldiazenyl)-2H-chromen-2-one (**6d**) 

Yields: 0.34 g. 76%. ^1^H NMR (DMSO-d_6_. 400 MHz). δ (ppm): 2.32 (s. 3H. CH_3_); 7.47 (s. 1H. CH); 7.61–7.67 (m. 4H. 4CH); 7.71 (d. 2H. 2CH. J = 8.4 Hz); 7.80 (d. 2H. 2CH. J = 8.4 Hz); 7.94 (d. 2H. 2CH. J = 7.0 Hz); 8.17 (dd. 1H. CH. J_1_ = 2.1 Hz. J_2_ = 9.1 Hz); 8.37 (s. 1H. CH); 8.45 (d. 1H. CH. J = 2.1 Hz); 11.49 (bs. 1H. NH). ^13^C NMR (DMSO-d_6_. 100 MHz); δ (ppm): 16.59 (C); 93.85 (C); 105.83 (C); 117.76 (C); 120.06 (C); 123.10 (2C); 124.38 (C); 125.83 (C); 127.76 (C); 128.14 (2C); 130.02 (2C); 132.25 (C); 134.68 (C); 137.86 (2C); 140.94 (C); 145.19 (C); 148.90 (C); 152.31 (C); 155.39 (C); 159.18 (C); 169.83 (C). ESI-HRMS (m/z) calculated for C_26_H_19_IN_5_O_2_S: 592.0304 [M+H]^+^. Found: 592.0299 [M+H]^+^. T_m_ = 233 °C.

#### 4.3.8. 3-(1-(2-(4-(3.4-Dichlorophenyl)thiazol-2-yl)hydrazono)ethyl)-6-(phenyldiazenyl)-2H-chromen-2-one (**6e**) 

Yields: 0.18 g. 56%. (dichloromethane/methanol (95:5). R_f_ = 0.78). ^1^H NMR (DMSO-d_6_. 400 MHz). δ (ppm): 2.33 (s. 3H. CH_3_); 2.35 (s. 3H. CH_3_); 7.25 (d. 1H. CH. J = 8.4 Hz); 7.32 (bs. 1H. CH); 7.61–7.68 (m. 4H. 4CH); 7.80 (d. 2H. 2CH. J = 7.7 Hz); 7.96 (d. 2H. 2CH. J = 7.7 Hz); 8.18 (dd. 1H. CH. J_1_ = 2.1 Hz. J_2_ = 8.4 Hz); 8.38 (s. 1H. CH); 8.48 (d. 1H. CH. J = 2.1 Hz); 11.42 (bs. 1H. NH). ESI-HRMS (m/z) calculated for C_26_H_18_Cl_2_N_5_O_2_S: 534.0558 [M+H]^+^. Found: 534.0560 [M+H]^+^. T_m_ = 195 °C.

#### 4.3.9. 3-(1-(2-(4-(4-Methoxyphenyl)thiazol-2-yl)hydrazono)ethyl)-6-(phenyldiazenyl)-2H-chromen-2-one (**6f**) 

Yields: 0.27 g. 67%. (dichloromethane/methanol (95:5). R_f_ = 0.73). ^1^H NMR (DMSO-d_6_. 400 MHz). δ (ppm): 2.32 (s. 3H. CH_3_); 3.81 (s. 3H. OCH_3_); 7.00 (d. 2H. 2CH. J = 8.4 Hz); 7.22 (s. 1H. CH); 7.61–7.67 (m. 4H. 4CH); 7.83 (d. 2H. 2CH. J = 8.4 Hz); 7.95 (d. 2H. 2CH. J = 8.4 Hz); 8.16 (dd. 1H. CH. J_1_ = 2.1 Hz. J_2_ = 8.4 Hz); 8.37 (s. 1H. CH); 8.46 (m. 1H. CH); 11.43 (bs. 1H. NH). ^13^C NMR (DMSO-d_6_. 100 MHz); δ (ppm): 16.60 (C); 55.63 (C); 102.78 (C); 114.49 (2C); 117.75 (C); 120.07 (C); 123.06 (2C); 124.39 (C); 125.78 (C); 127.40 (2C); 127.78 (C); 130.01 (2C); 132.22 (C); 140.91 (C); 145.19 (C); 148.88 (C); 150.31 (C); 152.30 (C); 155.37 (C); 159.21 (C); 159.35 (C); 169.68 (C). ESI-HRMS (m/z) calculated for C_27_H_22_N_5_O_3_S: 496.1443 [M+H]^+^. Found: 496.1438 [M+H]^+^. T_m_ = 231 °C.

#### 4.3.10. 6-(Phenyldiazenyl)-3-(1-(2-(4-p-tolylthiazol-2-yl)hydrazono)ethyl)-2H-chromen-2-one (**6g**)

Yields: 0.34 g. 83%. (dichloromethane/methanol (95:5). R_f_ = 0.88). ^1^H NMR (DMSO-d_6_. 400 MHz). δ (ppm): 2.34 (s. 3H. CH_3_); 7.62–7.69 (m. 4H. 4CH); 7.82 (s. 1H. CH); 7.96 (d. 2H. 2CH. J = 7.3 Hz); 8.16–8.20 (m. 3H. 3CH); 8.32 (d. 2H. 2CH. J = 9.8 Hz); 8.41 (s. 1H. CH); 8.48 (d. 1H. CH. J = 2.1 Hz); 11.63 (s. 1H. NH). ^13^C NMR (DMSO-d_6_, 100 MHz); δ (ppm): 16.58 (C); 21.27 (C); 103.97 (C); 117.76 (2C); 120.13 (C); 123.05 (2C); 124.41 (C); 125.77 (C); 126.01 (2C); 127.84 (C); 129.62 (2C); 129.99 (2C); 132.21 (C); 132.41 (C); 137.28 (C); 140.91 (C); 15.09 (C); 148.88 (C); 152.34 (C); 155.39 (C); 159.21 (C); 169.69 (C). ESI-HRMS (m/z) calculated for C_27_H_22_N_5_O_2_S: 480.1494 [M+H]^+^. Found: 480.1490 [M+H]^+^. T_m_ = 232 °C.

#### 4.3.11. 3-(1-(2-(4-(4-Nitrophenyl)thiazol-2-yl)hydrazono)ethyl)-6-(phenyldiazenyl)-2H-chromen-2-one (**6h**) 

Yields: 0.26 g. 51%. (dichloromethane/methanol (95:5). R_f_ = 0.71). ^1^H NMR (DMSO-d_6_. 400 MHz). δ (ppm): 1.66–1.75 (m. 6H. 3CH_2_); 1.88 (bs. 6H. 3CH_2_); 2.02 (bs. 3H. 3CH); 2.26 (s. 3H. CH3); 6.36 (s. 1H. CH); 7.58–7.64 (m. 4H. 4CH); 7.90–7.94 (m. 2H. 2CH); 8.13 (dd. 1H. CH. J_1_ = 2.1 Hz. J_2_ = 8.4 Hz); 8.29 (s. 1H. CH); 8.42 (d. 1H. CH. J = 2.1 Hz); 11.23 (bs. 1H. NH). ESI-HRMS (m/z) calculated for C_26_H_19_N_6_O_4_S: 511.1188 [M+H]^+^. Found: 511.1187 [M+H]^+^. Melting with degradation at 256 °C.

#### 4.3.12. 3-(1-(2-(4-(1-Adamantyl)thiazol-2-yl)hydrazono)ethyl)-6-(phenyldiazenyl)-2H-chromen-2-one (**6i**) 

Yields: 0.29 g. 68%. (dichloromethane/methanol (95:5). R_f_ = 0.83). ^1^H NMR (DMSO-d_6_. 400 MHz). δ (ppm): 2.31 (s. 3H. CH_3_); 7.60–7.71 (m. 7H. 7CH); 7.86–7.90 (m. 1H. CH); 7.92–7.96 (m. 2H. 2CH); 8.13–8.18 (m. 2H. 2CH); 8.37 (s. 1H. CH); 8.45 (d. 1H. CH. J = 3.2 Hz); 11.50 (s. 1H. NH). ^13^C NMR (DMSO-d6. 100 MHz); δ (ppm): 16.67 (C); 28.33 (6C); 36.27 (3C); 36.75 (3C); 41.58 (C); 100.94 (C); 117.76 (C); 120.04 (C); 123.07 (2C); 124.37 (C); 125.81 (C); 127.78 (C); 130.00 (C); 132.25 (C); 140.98 (C); 148.88 (C); 152.32 (C); 155.39 (C); 159.18 (C); 169.72 (C). ESI-HRMS (m/z) calculated for C_30_H_30_N_5_O_2_S: 524.2120 [M+H]^+^. Found: 524.2120 [M+H]^+^. T_m_ = 239 °C.

### 4.4. Guest-Host Azopolymer Films Preparation

Guest-host systems were prepared by dissolving the polystyrene matrix and the required amount of azocumarin (**6a**–**6f**) in NMP. The concentration of azocompound was 5 wt.%. Polymer films on the glass substrates for *cis*-*trans* isomerization process investigations were prepared as follows: the homogeneous polymers solutions were obtained by dissolving polymer powder in NMP solvent. Then solutions were filtered through 0.02 µm membranes and cast onto clean glass substrates. To remove the residual solvent, the films were initially heated at 60 °C until the solid-state was achieved and then dried under vacuum at 80 °C for 2 days.

### 4.5. UV-Vis Measurements

The measurements of the UV-Vis absorption spectra were carried out with a Jasco V750-FLH740 spectrophotometer using a 1 cm optical path quartz cuvette with ca. 1 × 10^−5^ mol/L solutions of azocumarin derivative in chloroform, ethanol, DMF and in PS. A population of *cis*-isomers was generated by irradiating the sample solutions with UV light (λ = 365 nm) from a 2.9 W diode for 30 s. The conversion efficiency of the trans-isomer transition into the *cis*-isomer was determined by monitoring the change in the absorbance at the wavelength corresponding to the absorption maximum of the trans-form, before and immediately after light irradiation. Content of *cis*-isomer in the photostationary state was calculated according to the following equation [22]:

(1) $$P=\frac{A_{0}-A_{t}}{A_{0}}\;$$

where *A*_0_ and *A_t_* correspond to the normalized maximum of absorbance before and after 30-s irradiation, respectively.

### 4.6. Solar Cell Preparation

Photovoltaic devices with the bulk-heterojunction (BHJ) structure were prepared on the ITO-coated glass substrates (Ossila Ltd. Sheffield. UK. 6 pixels. each with an area of 4.5 mm^2^). The substrates were cleaned with surfactant (Hellmanex III. Hellma Analytics) and subsequently with isopropanol in the ultrasonic bath. Thin-film of PEDOT:PSS was obtained by spin-coated on ITO electrodes at 5000 rpm. The PEDOT:PSS thin films were annealed at 120 °C for 10 min. Solutions of the active layer were prepared by dissolving blends of azo-compounds and fullerene derivatives in chlorobenzene using a weight ratio of 1:5. Such prepared solutions were deposited on the PEDOT:PSS layers by spin-coating at 1000 rpm and subsequently. annealed at 120 °C for 10 min. An aluminium counter electrode was evaporated on the top of the active layer and current density-voltage (J-V) characteristics have been registered using PV Test Solutions Solar Simulator (PV Solutions, Poland). under the solar illumination of AM1.5 standard using Keithley 2400 electrometer. Tektronix, Inc., Beaverton, OR, USA

## Figures and Tables

**Table 1:** Electrochemical data of the investigated azodyes (0.1M Bu_4_NBF_4_/CH_2_Cl_2_ solution).

Code	E_ox_^onset^ [V]	E_red_ ^onset^ [V]	HOMO [eV]	LUMO [eV]	E_g_ ^CV^ [eV]	E_g_ ^OPT^ [eV]
**6a**	0.38	−1.75	−5.48	−3.35	2.13	2.40
**6b**	0.33	−1.42	−5.43	−3.68	1.75	2.36
**6c**	0.25	−1.48	−5.35	−3.62	1.73	2.20
**6d**	0.25	−1.46	−5.35	−3.64	1.71	2.22
**6e**	0.51	−1.31	−5.61	−3.79	1.82	2.34
**6f**	0.19	−1.52	−5.29	−3.58	1.71	2.20
**6g**	0.31	−1.53	−5.41	−3.57	1.84	2.22
**6h**	−	−1.32	−	−3.79	−	2.44
**6i**	0.35	−1.60	−5.45	−3.50	1.95	2.18
HOMO = −5.1 – E_ox_ ^onset^. LUMO = −5.1 − E_red_ ^onset^, E_g_ ^CV^ = E _ox_ ^onset^ – E _red_ ^onset^, E_g_ ^OPT^ = 1240/λ						

**Table 2:** The values of absorption maximum (λ_max_) and molar absorption coefficient (ε) in chloroform, ethanol, and DMF (c =10^−5^ mol/L).

Code	Solvent					
	CHCl_3_		C_2_H_5_OH		DMF	
	λ_max_ [nm]	ε [dm^3^mol^−1^cm^−1^]	λ_max_ [nm]	ε [dm^3^mol^−1^cm^−1^]	λ_max_ [nm]	ε [dm^3^mol^−1^cm^−1^]
**6a**	290 337	29,999 29,392	289	29,400	285	27,700
**6b**	291 338	24,200 19,450	284	31,500	286	37,400
**6c**	292 335	30,475 23,718	292	33,600	282	38,900
**6d**	294 334	30,900 21,700	288	37,700	283	36,700
**6e**	293 340	32,800 21,500	–*	–*	288	38,100
**6f**	293 330	39,250 30,800	281	38,150	281	35,016
**6g**	290 340	32,500 27,100	248 288	33,000 36,700	283	13,100
**6h**	296 344	31,684 28,185	–*	–*	298 346	15,600 12,000
**6i**	289 336	26,500 30,700	296 326	33,450 33,400	292 336	38,500 29,400

* sample insoluble in ethanol.

**Table 3:** Content of *cis*-isomer directly after the 30 s of UV-light irradiation, and time of *cis* to *trans* transformation of dyes in chloroform, ethanol, and DMF.

Code	Solvent								
	CHCl_3_			C_2_H_5_OH			DMF		
	Isosbestic Points [nm]	Content of *cis* Isomer (%)	Time of *cis-trans* Conversion [h]	Isosbestic Points [nm]	Content of *cis* Isomer (%)	Time of *cis-trans* Conversion [days]	Isosbestic Points [nm]	Content of *cis* Isomer (%)	Time of *cis-trans* Conversion [Days]
**6a**	285; 416	16	24	291; 420	13.5	>>4	282; 428	8	>7
**6b**	286; 415	11	25	275; 418	10	>4	423	6	>7
**6c**	286; 417	16	29	279; 421	11.5	>>4	286; 426	7	>7
**6d**	286; 421	15	28	278; 418	10	4	279; 430	7	>7
**6e**	279; 432	13	>>24	–*	–*	–*	281; 416	9	>>7
**6f**	286; 414	22	>>27	281; 425	10	>>4	281; 428	8	>7
**6g**	287; 421	14	24	279; 434	10	>>4	283; 425	11	>7
**6h**	286; 419	7	24	–*	–*	–*	282; 419	6.5	>7
**6i**	285; 415	14	24	292; 394	13	>>4	282; 420	7	>7

* sample insoluble in ethanol.

**Table 4:** PV parameters of fabricated solar cells.

Solar Cell Architecture	V_OC_ [mV]	J_SC_ [mA/cm^2^]	FF	PCE [%]	R_SH_ [kΩ]	R_S_ [kΩ]
ITO/PEDOT:PSS/**6a**:PC_70_BM/Al	no activity detected				1.15 ± 0.69	1.31 ± 0.90
ITO/PEDOT:PSS/**6b**:PC_70_BM/Al	381 ± 58	0.53 ± 0.05	0.32 ± 0.02	0.07 ± 0.02	24.71 ± 14.62	8.73 ± 0.73
ITO/PEDOT:PSS/**6c**:PC_70_BM/Al	303 ± 70	0.44 ± 0.04	0.32 ± 0.02	0.05 ± 0.01	16.29 ± 5.59	8.67 ± 0.94
ITO/PEDOT:PSS/**6d**:PC_70_BM/Al	375 ± 39	0.61 ± 0.00	0.34 ± 0.02	0.08 ± 0.01	22.67 ± 0.81	6.11 ± 0.42
ITO/PEDOT:PSS/**6e**:PC_70_BM/Al	no activity detected				10.53 ± 2.59	12.07 ± 2.54
ITO/PEDOT:PSS/**6f**:PC_70_BM/Al	367 ± 71	0.47 ± 0.04	0.35 ± 0.03	0.07 ± 0.02	26.96 ± 11.52	7.38 ± 0.98
ITO/PEDOT:PSS/**6g**:PC_70_BM/Al	420 ± 87	0.63 ± 0.06	0.34 ± 0.03	0.10 ± 0.03	24.63 ± 10.17	6.23 ± 1.69
ITO/PEDOT:PSS/**6h**:PC_70_BM/Al	496 ± 85	0.43 ± 0.03	0.32 ± 0.02	0.07 ± 0.02	37.77 ± 7.81	10.89 ± 2.51
ITO/PEDOT:PSS/**6i**:PC_70_BM/Al	no activity detected				7.67 ± 2.90	9.10 ± 2.67
ITO/PEDOT:PSS/PTB7:PC_70_BM:**6g**/PFN/Al (4:8:1)	611 ± 8	7.16 ± 0.70	0.45 ± 0.01	2.01 ± 0.24	6.39 ± 1.75	0.54 ± 0.03
ITO/PEDOT:PSS/PTB7:PC_70_BM:**6g**/PFN/Al (13:8:1)	634 ± 7	10.80 ± 0.50	0.38 ± 0.02	2.68 ± 0.11	2.98 ± 0.59	0.58 ± 0.04

## Data Availability

Not applicable.

## Supplementary Materials

The following supporting information can be downloaded at: https://www.mdpi.com/article/10.3390/ijms23105767/s1.
