# Supplementary material for: Novel Azocoumarin Derivatives—Synthesis and Characterization
Source: Int J Mol Sci. 2022 May 21;23(10):5767. doi: 10.3390/ijms23105767 (PMC9147163; doi:10.3390/ijms23105767)
Supplement: Supplementary file 1 [file ijms-23-05767-s001.zip › ijms-1714144-supplementary.pdf]

Supporting Information

for

Novel azocoumarin derivatives synthesis and characterization

Katarzyna Piechowska[1], Angelika Baranowska-Łączkowska[2], Krzysztof Z. Łączkowski[1], Jolanta Konieczkowska[3], Mariola Siwy[3], Marharyta Vasylieva[3], Paweł Gnida[3], Paweł Nitschke[3], Ewa Schab-Balcerzak[3]

[1]Department of Chemical Technology and Pharmaceuticals, Faculty of Pharmacy, Collegium Medicum, Nicolaus Copernicus University, Jurasza 2, 85-089 Bydgoszcz, Poland; kpiechowska@cm.umk.pl (K.P); krzysztof.laczkowski@cm.umk.pl (K.Z.L.)

[2]Institute of Physics, Kazimierz Wielki University, Powstańców Wielkopolskich 2, 85-090, Bydgoszcz, Poland; angelika.baranowska@ukw.edu.pl (A.B.L.)

[3]Centre of Polymer and Carbon Materials, Polish Academy of Sciences, 34 M. Curie-Skłodowska Str., 41-819 Zabrze, Poland

Table S1. Solubility of azocumarin derivatives in different solvents (2 mg of the compound in 1ml of solvent).

Sample	H_2O		THF		$CHCl_3$		DMF		CH_3OH	
	room temp.	boiling temp.	room temp.	boiling temp.	room temp.	boiling temp.	room temp.	boiling temp.	room temp.	boiling temp.
**6-a**	–	+/-	+/-	+/-	+/-	+/-	+	+	+/-	+/-
**6-b**	–	–	+/-	+/-	+/-	+/-	+	+	+/-	+/-
**6-c**	–	+/-	+/-	+/-	+/-	+/-	+	+	+/-	+/-
**6-d**	–	+/-	+/-	+/-	+/-	+/-	+	+	+/-	+/-
**6-e**	–	–	+/-	+/-	+/-	+/-	+	+	+/-	+/-
**6-f**	–	–	+/-	+/-	+/-	+/-	+/-	+	+/-	+/-
**6-g**	+/-	+/-	+/-	+/-	+/-	+/-	+/-	+	+/-	+/-
**6-h**	–	+/-	+/-	+/-	+	+	+/-	+	+/-	+/-
**6-i**	–	+/-	+/-	+/-	+	+	+/-	+	+/-	+/-

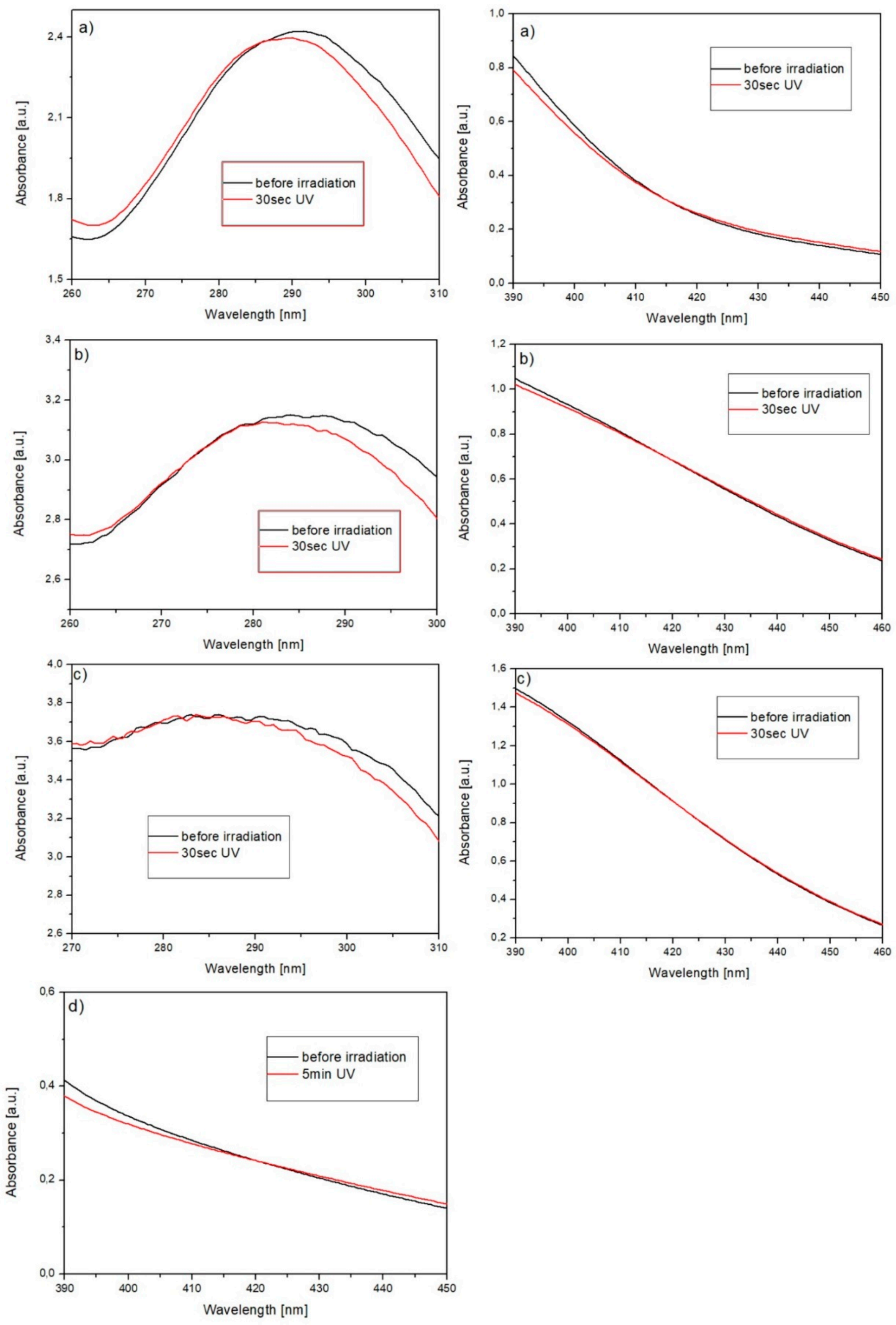

Figure S1. UV-vis spectra of **6b** registered before and after irradiation (λ=365nm, 2.9 W, 30s) in (a) chloroform, (b) ethanol, (c) DMF, and (d) polystyrene.

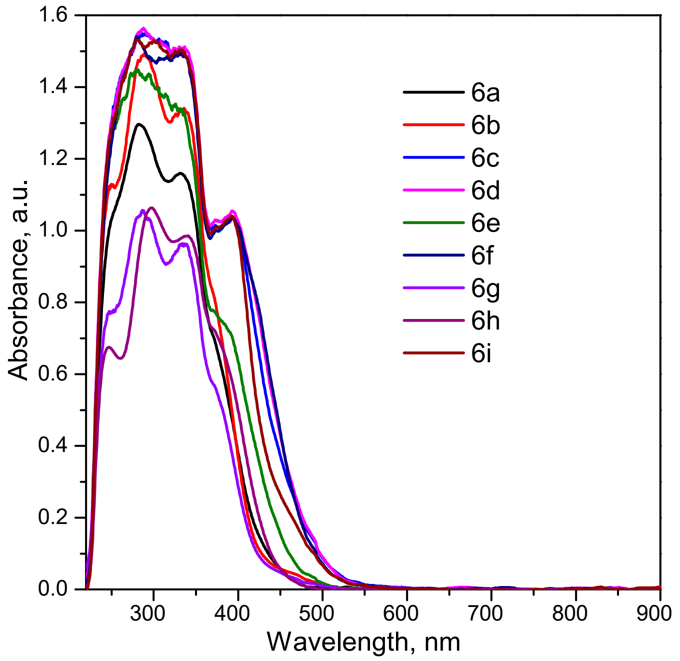

Figure S2. Absorbance spectra of compound 6a-6i in DCM

Table S2. Content of *cis*-isomer directly after the 5 min of UV-light irradiation, and time of *cis* to *trans* transformation of dyes in polystyrene matrix.

Code	Isosbestic points [nm]	Content of *cis* isomer (%)	Time of *cis*-*trans* conversion [days]
**6a+PS**	418	13	8
**6b+PS**	420	14	8
**6c+PS**	421	13	>8
**6d+PS**	424	11	>8
**6f+PS**	419	14	7
**6g+PS**	410	14	>8
**6h+PS**	437	7	7
**6i+PS**	407	22	>8

^{1}H, ^{13}C NMR and HRMS spectra of compounds **3-5**, **6a-6i** and additional DEPT-135, COSY, NOESY, HMBC, HSQC spectra for compound **6g**.

Compound 3

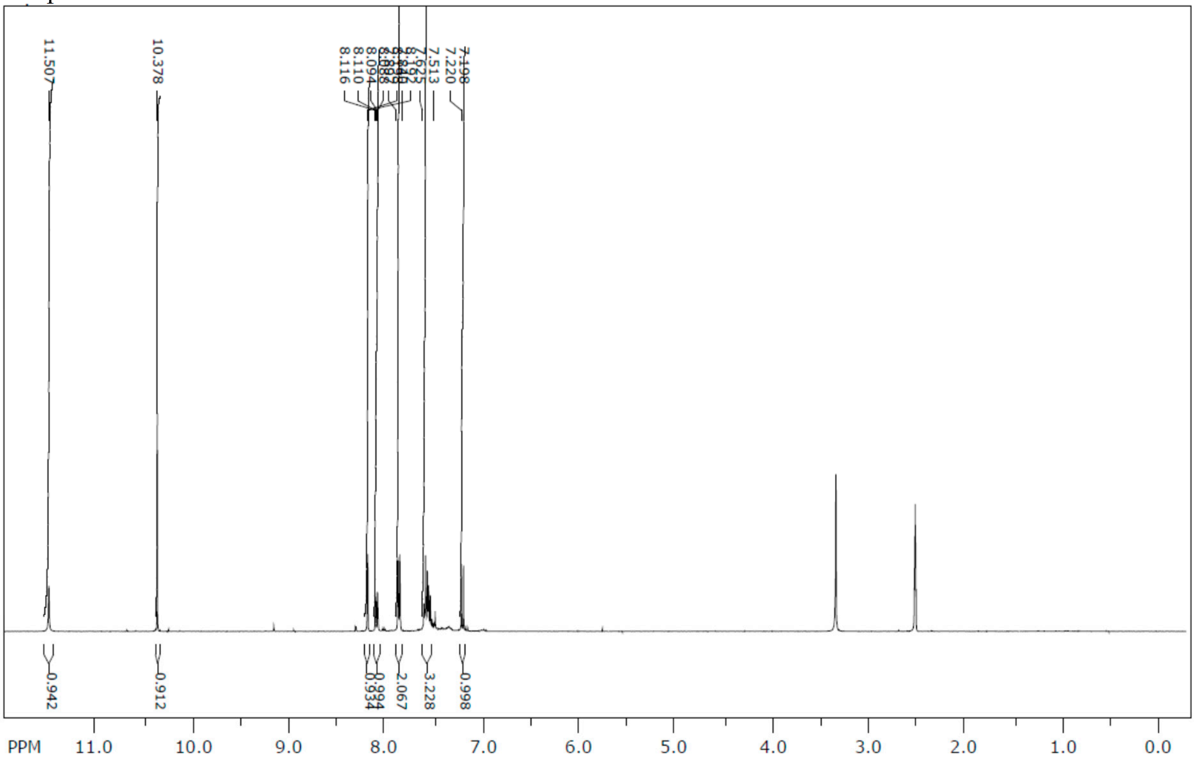

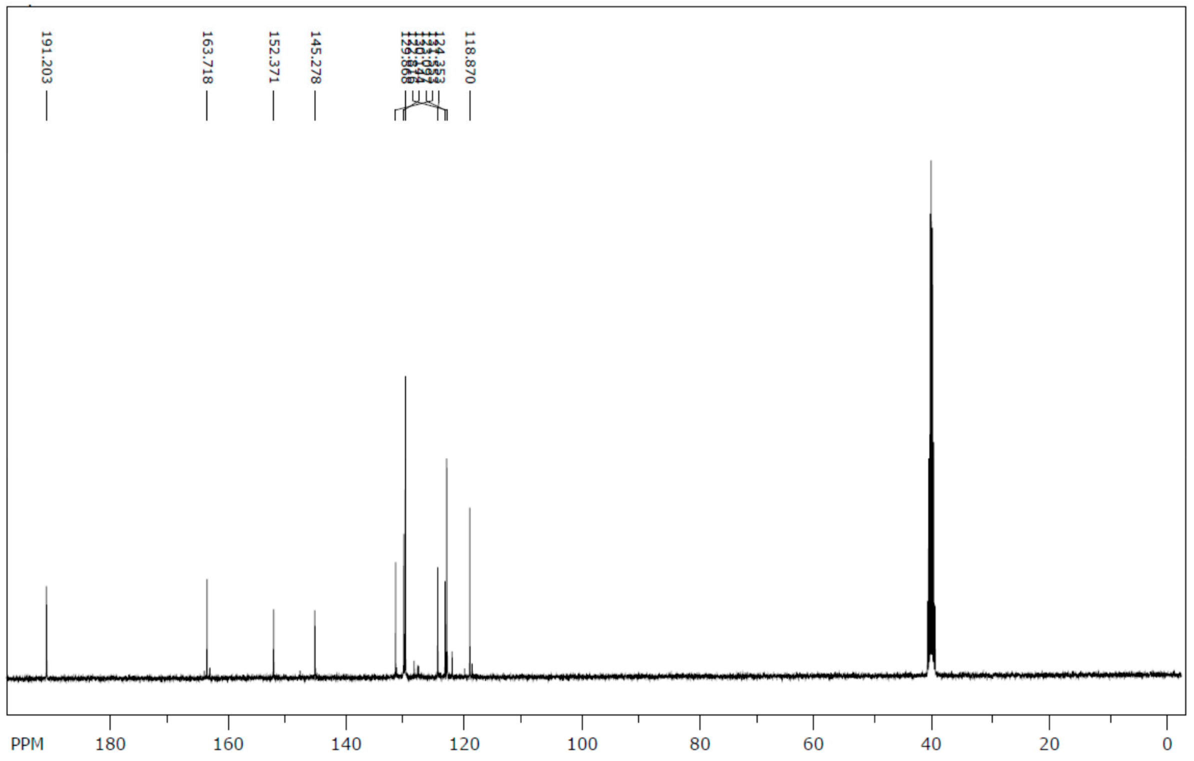

Compound 4

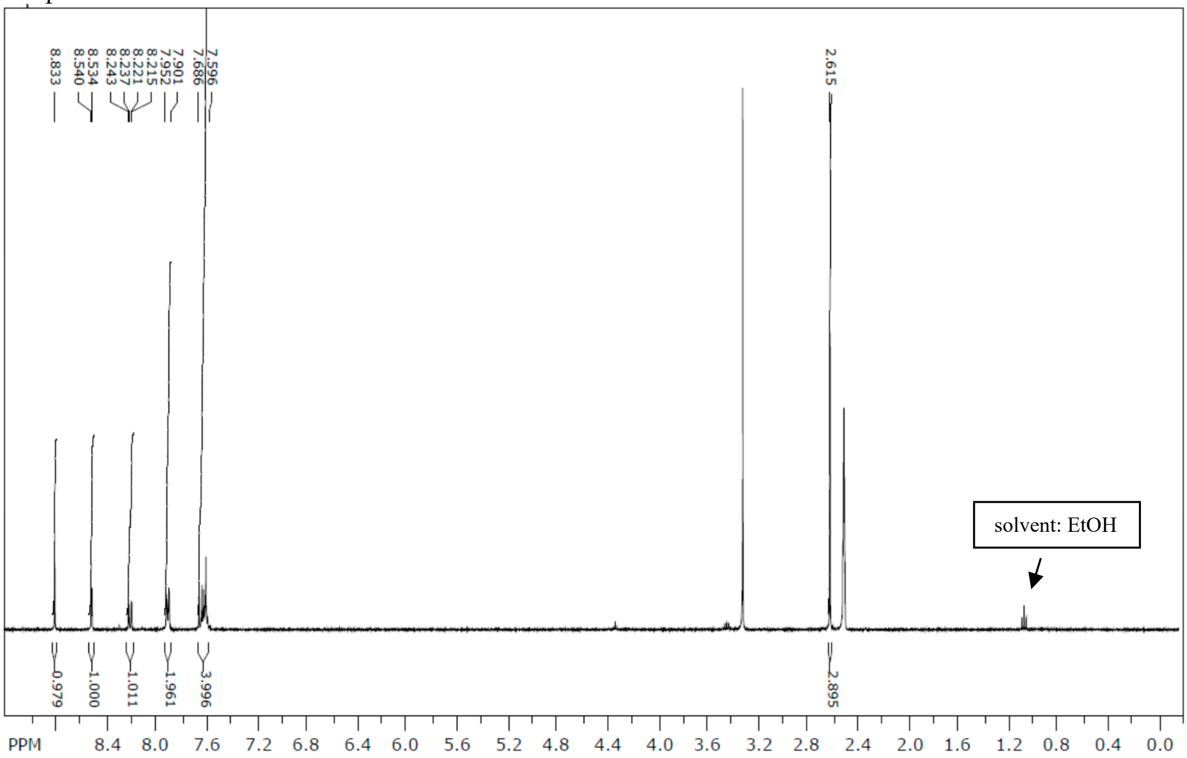

8.833
8.540
8.534
8.243
8.237
8.221
8.215
7.952
7.901
7.686
7.596
2.615
solvent: EtOH
0.979
1.000
1.011
1.961
3.996
2.895
PPM
8.4
8.0
7.6
7.2
6.8
6.4
6.0
5.6
5.2
4.8
4.4
4.0
3.6
3.2
2.8
2.4
2.0
1.6
1.2
0.8
0.4
0.0

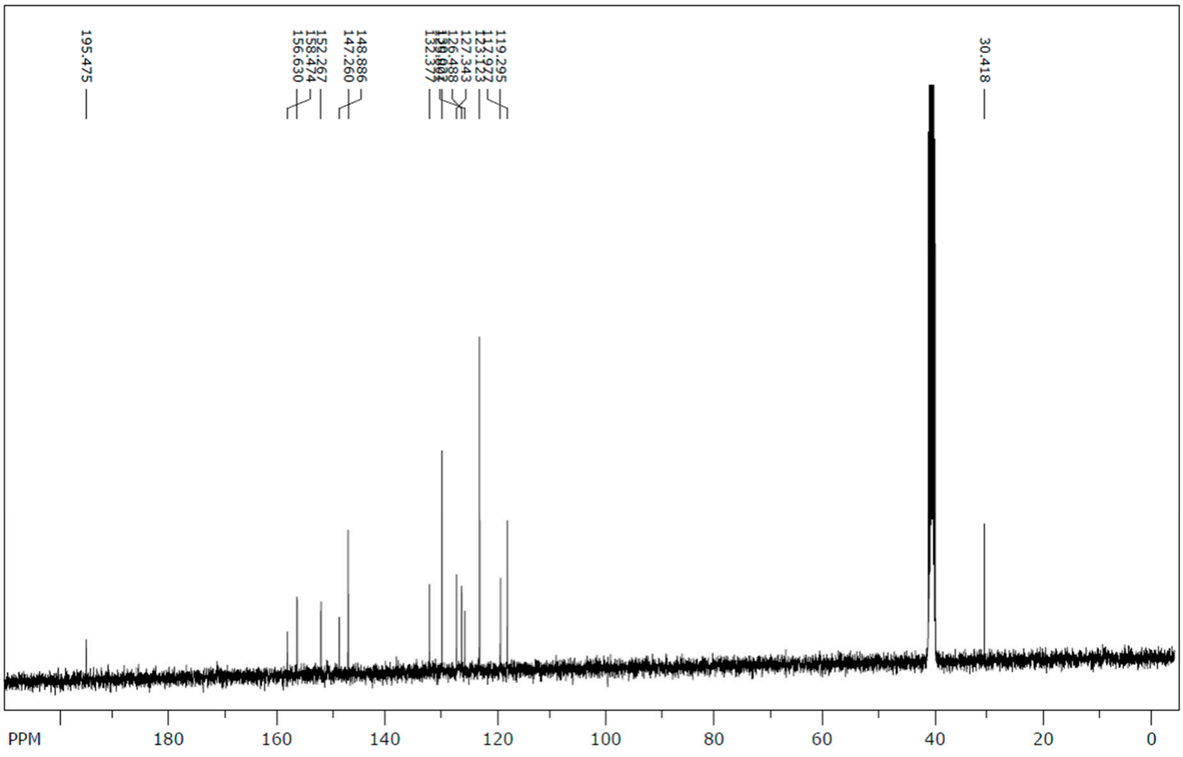

195.475
158.474
156.630
152.267
148.886
147.260
132.377
127.343
126.488
123.123
119.295
117.972
30.418
PPM
180
160
140
120
100
80
60
40
20
0

Compound 5

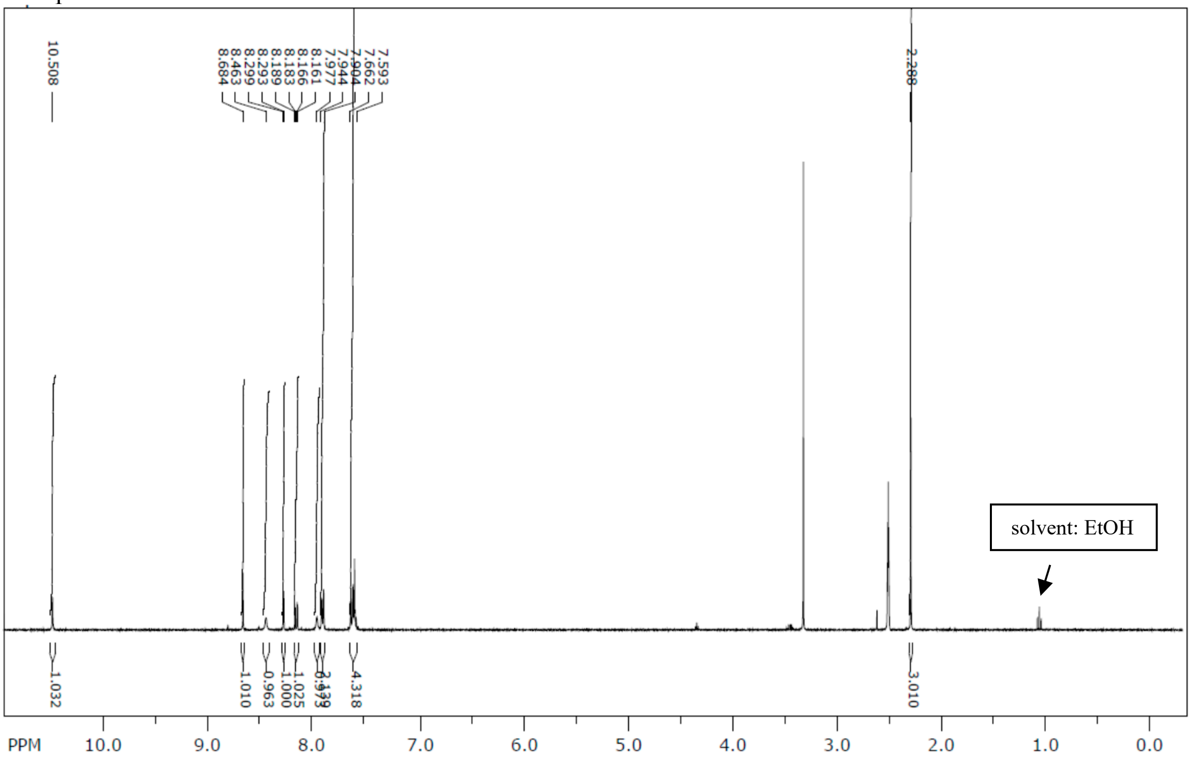

10.508
8.684
8.463
8.299
8.293
8.189
8.183
8.166
8.161
7.977
7.944
7.904
7.662
7.593
2.288
solvent: EtOH
1.032
1.010
0.963
1.000
1.025
4.318
3.010
PPM
10.0
9.0
8.0
7.0
6.0
5.0
4.0
3.0
2.0
1.0
0.0

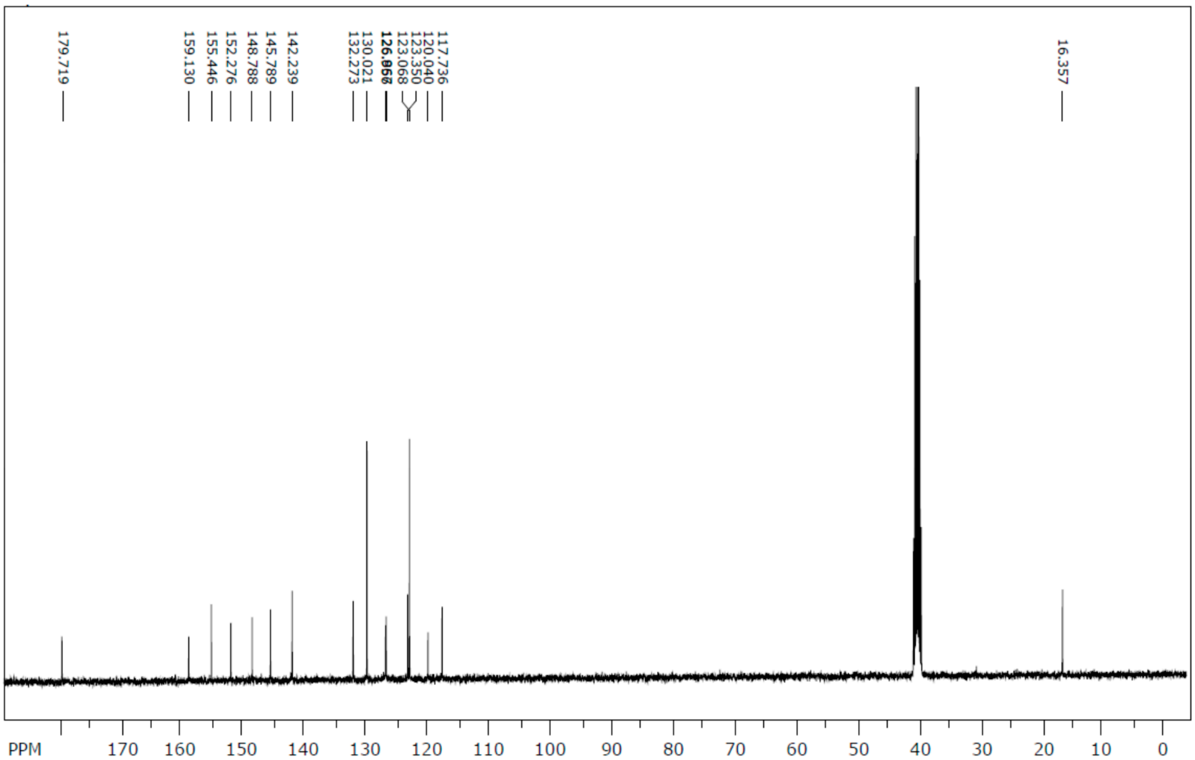

179.719
159.130
155.446
152.276
148.788
145.789
142.239
132.273
130.021
123.068
123.350
120.040
117.736
16.357
PPM
170
160
150
140
130
120
110
100
90
80
70
60
50
40
30
20
10
0

Compound 6a

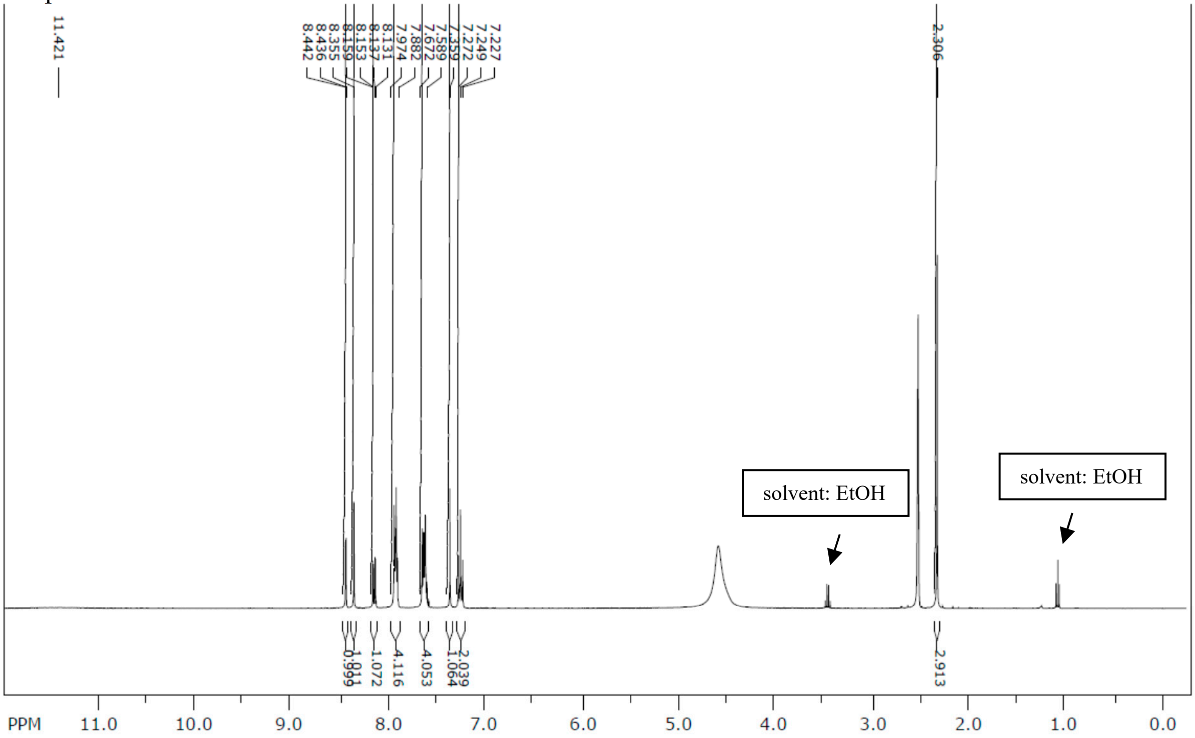

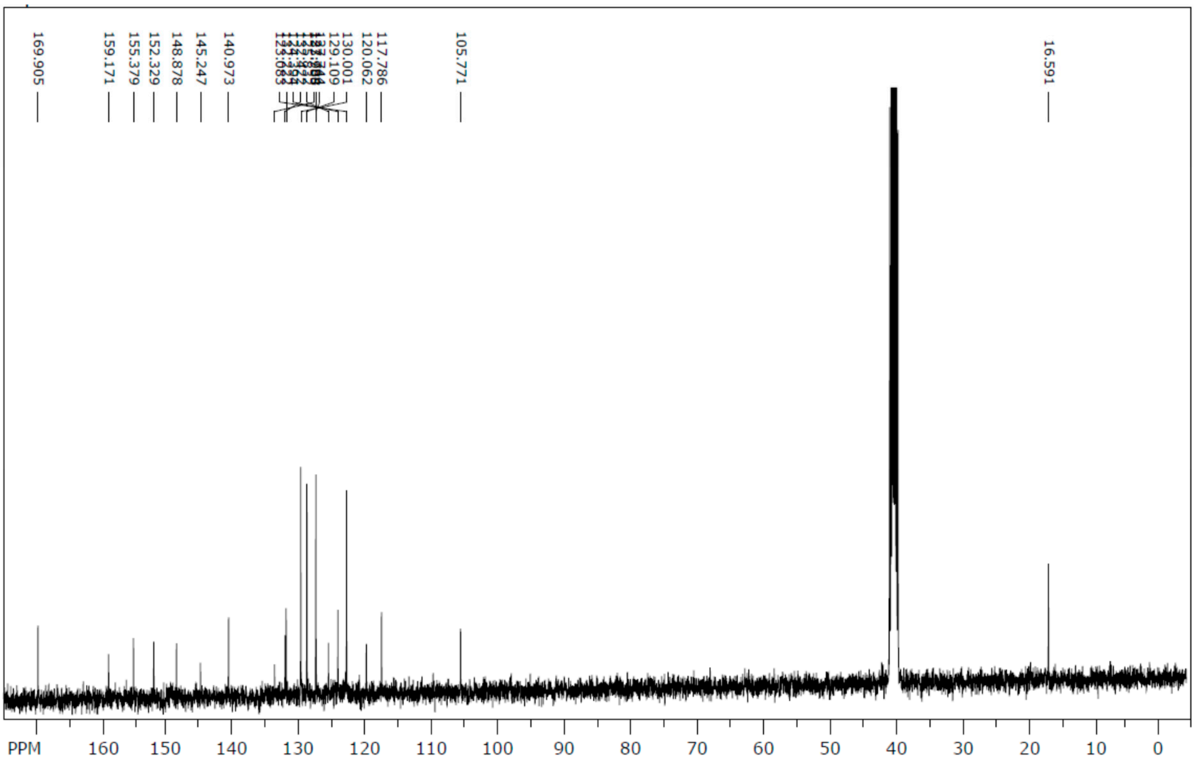

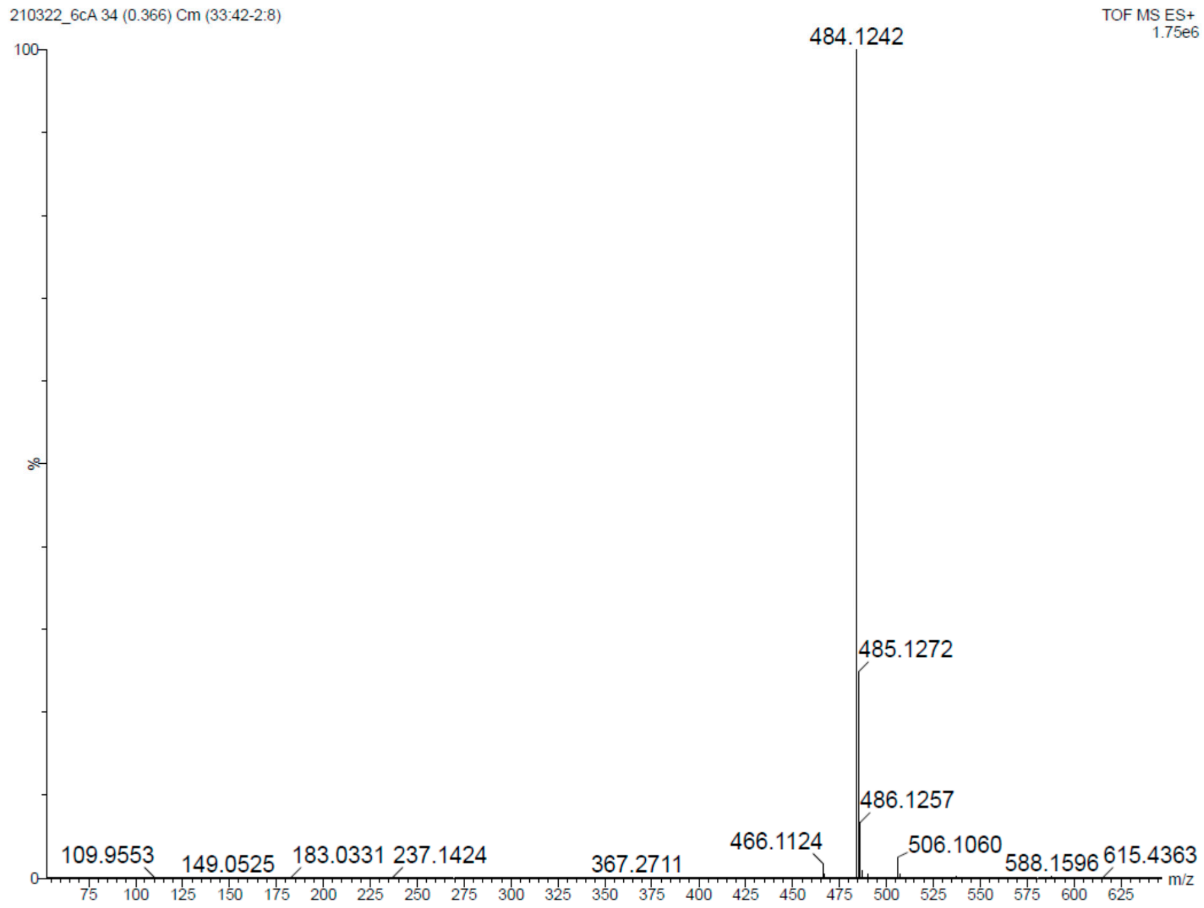
210322_6cA 34 (0.366) Cm (33:42-2:8)
TOF MS ES+
1.75e6
484.1242
485.1272
486.1257
466.1124
506.1060
588.1596
615.4363
109.9553
149.0525
183.0331
237.1424
367.2711
100
%
0
m/z

Compound 6b

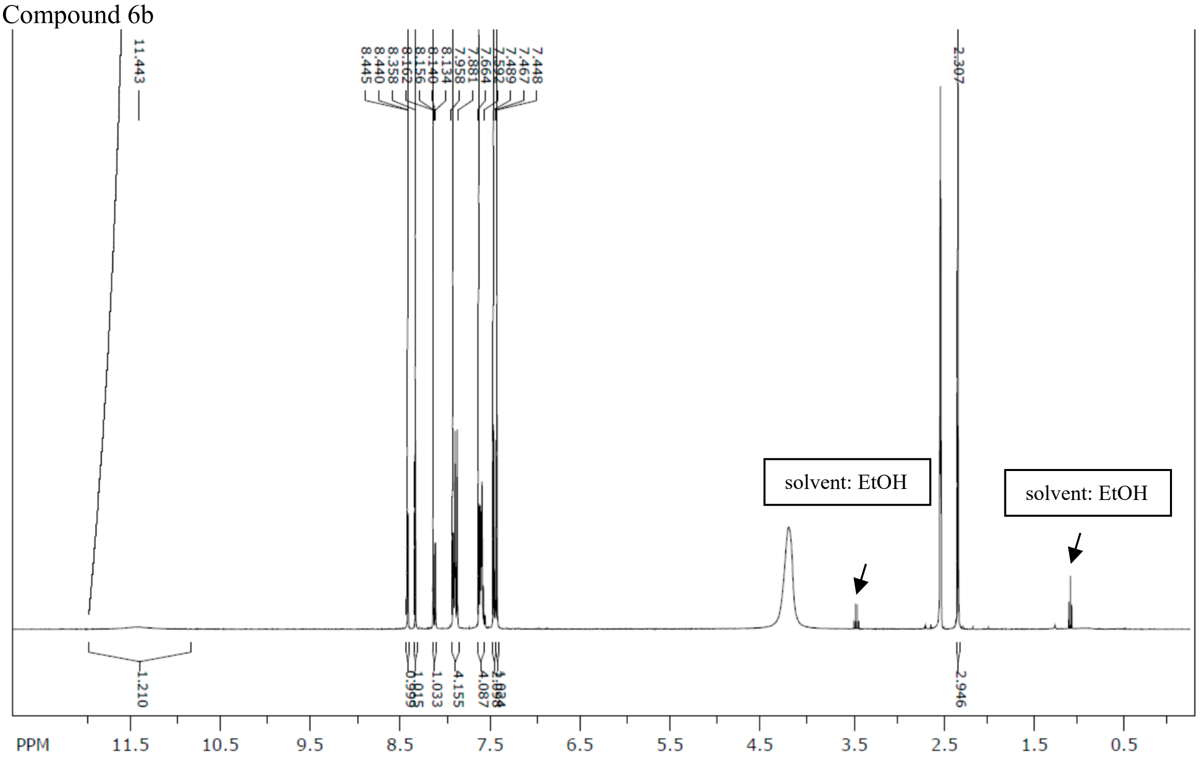
11.443
8.445
8.440
8.358
8.162
8.156
8.140
8.134
7.958
7.881
7.664
7.467
7.489
7.448
2.307
solvent: EtOH
solvent: EtOH
1.210
1.033
4.155
4.087
2.946
PPM

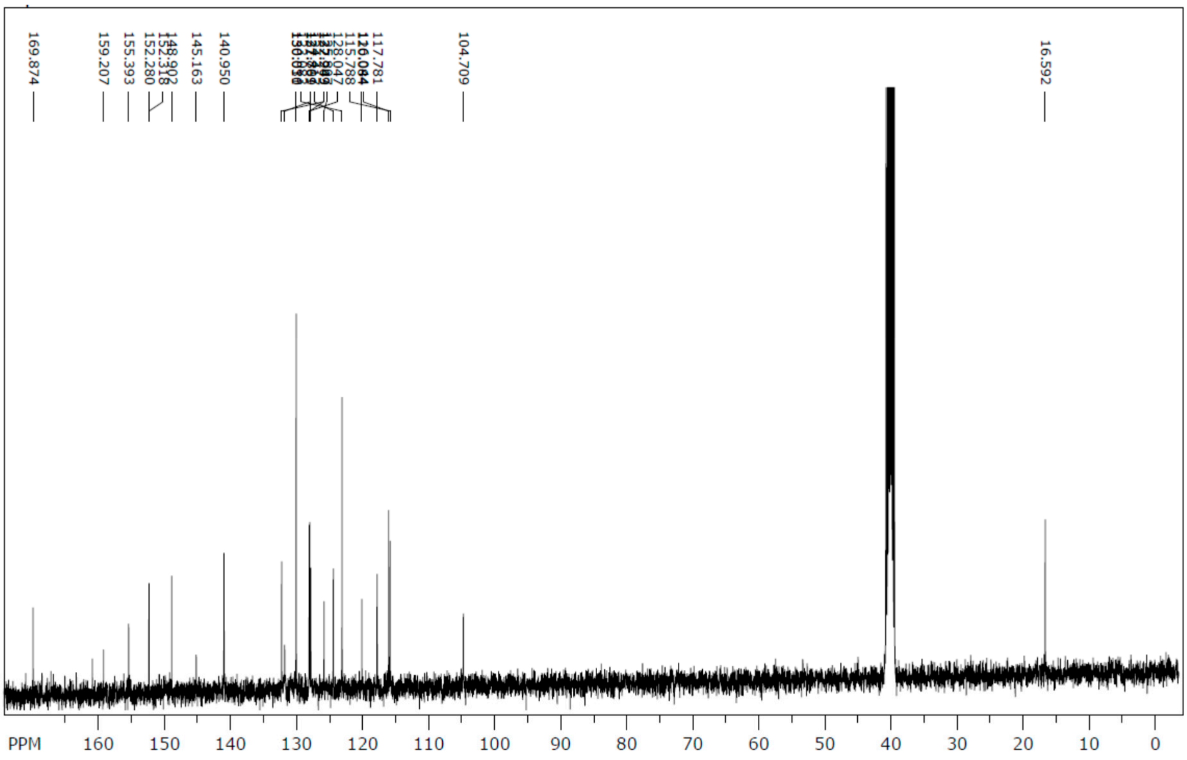
169.874
159.207
155.393
152.280
145.163
140.950
117.781
115.788
104.709
16.592
PPM
160
150
140
130
120
110
100
90
80
70
60
50
40
30
20
10
0

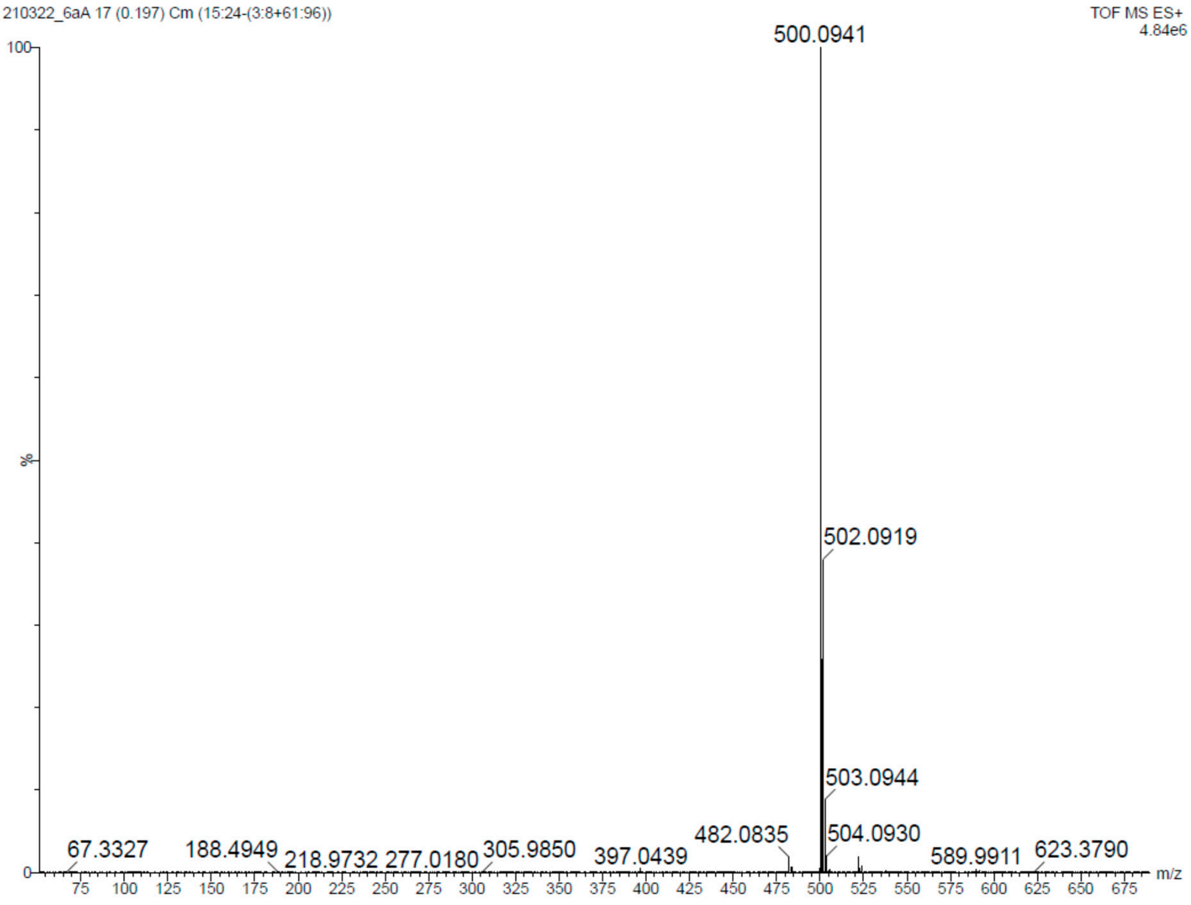
210322_6aA 17 (0.197) Cm (15:24-(3:8+61:96))
TOF MS ES+
4.84e6
500.0941
502.0919
503.0944
504.0930
482.0835
67.3327
188.4949
218.9732
277.0180
305.9850
397.0439
589.9911
623.3790
100
%
0
m/z

Compound 6c

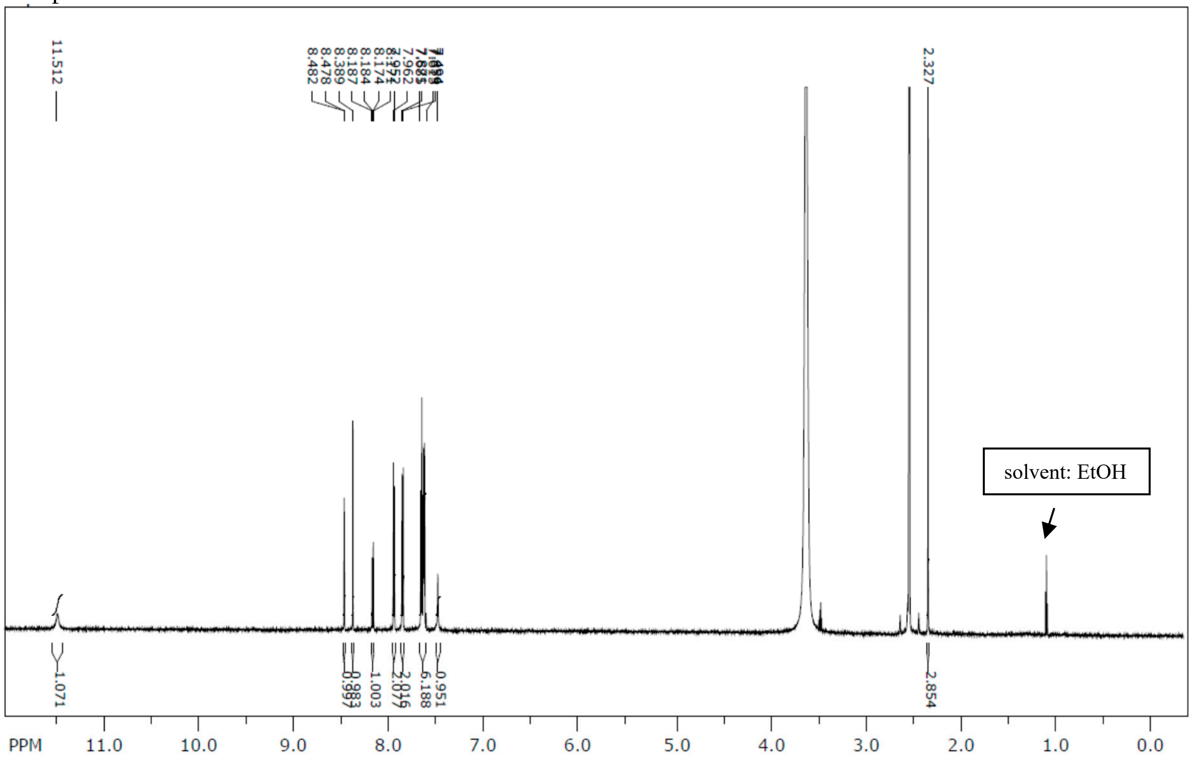

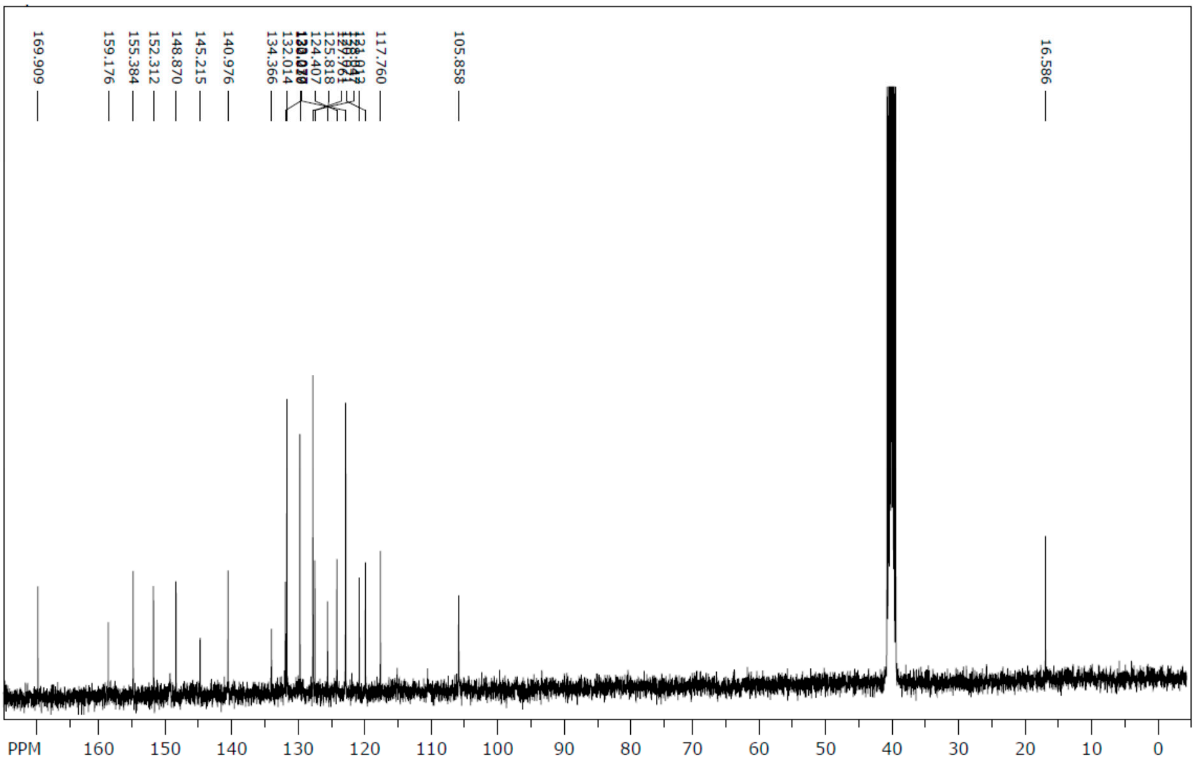

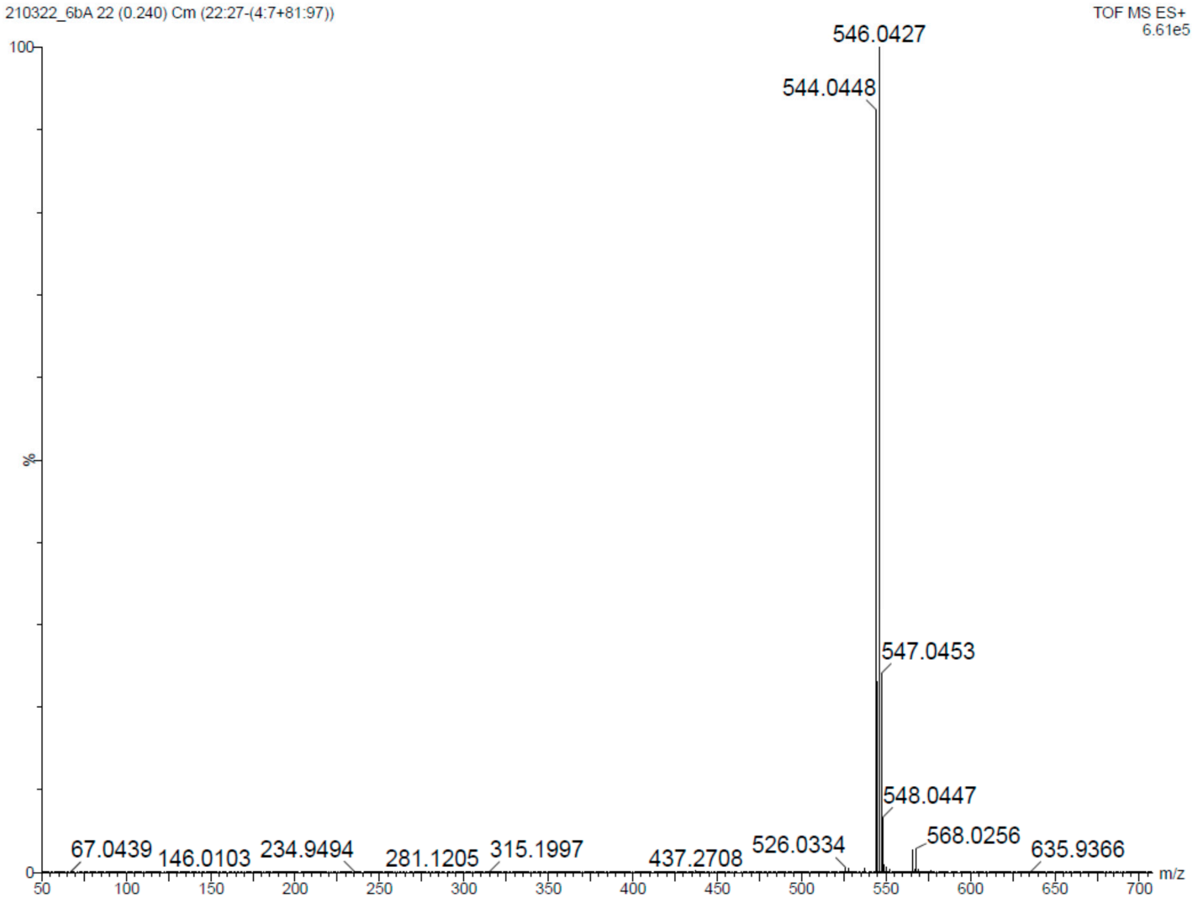

210322_6bA 22 (0.240) Cm (22:27-(4:7+81:97))
TOF MS ES+
6.61e5
546.0427
544.0448
547.0453
548.0447
568.0256
635.9366
526.0334
437.2708
315.1997
281.1205
234.9494
146.0103
67.0439
100
%
0
m/z

Compound 6d

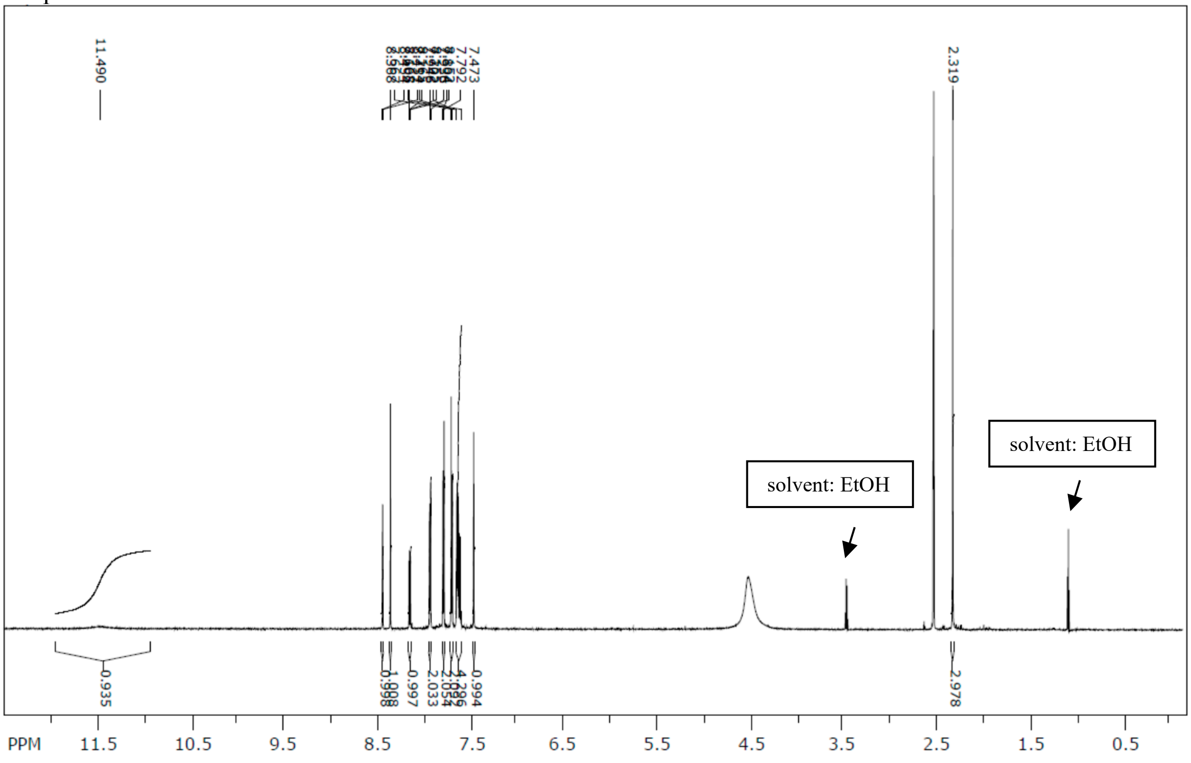

11.490
7.473
7.792
2.319
solvent: EtOH
solvent: EtOH
0.935
0.997
2.033
0.994
2.978
PPM
11.5
10.5
9.5
8.5
7.5
6.5
5.5
4.5
3.5
2.5
1.5
0.5

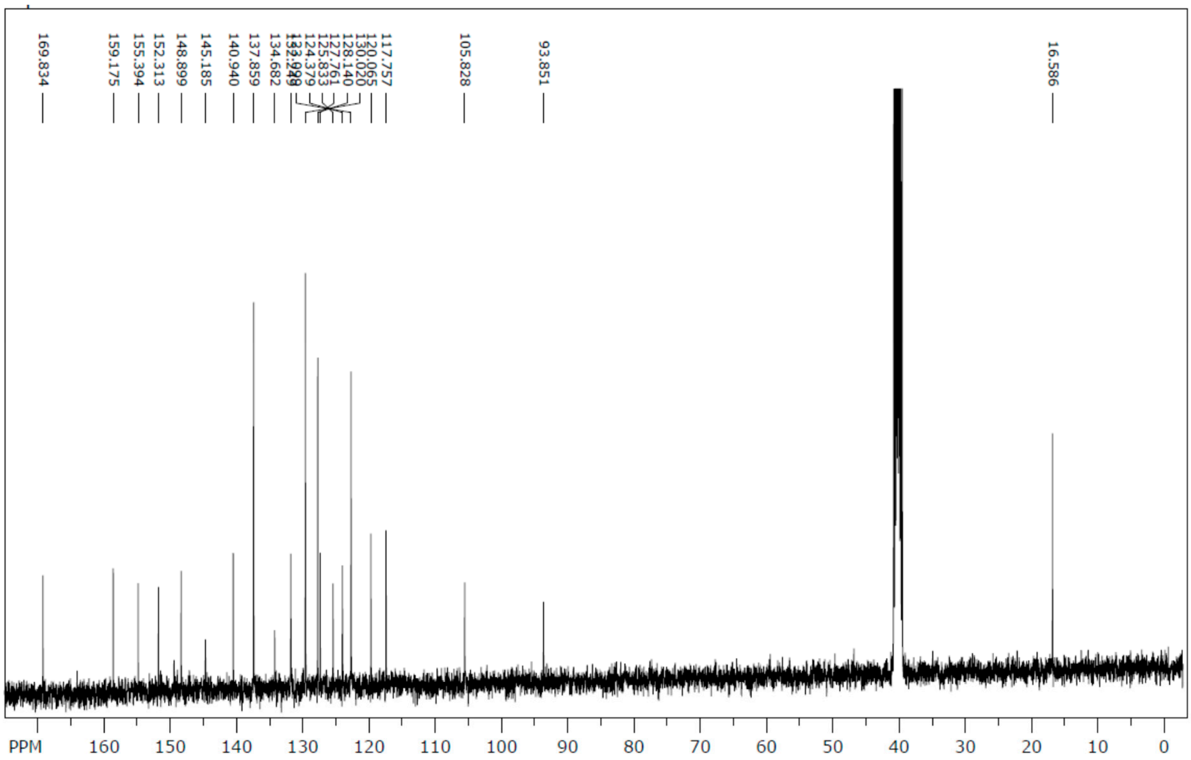
169.834
159.175
155.394
152.313
148.899
145.185
140.940
137.859
134.682
124.379
125.833
127.761
128.140
130.020
120.065
117.757
105.828
93.851
16.586
PPM 160 150 140 130 120 110 100 90 80 70 60 50 40 30 20 10 0

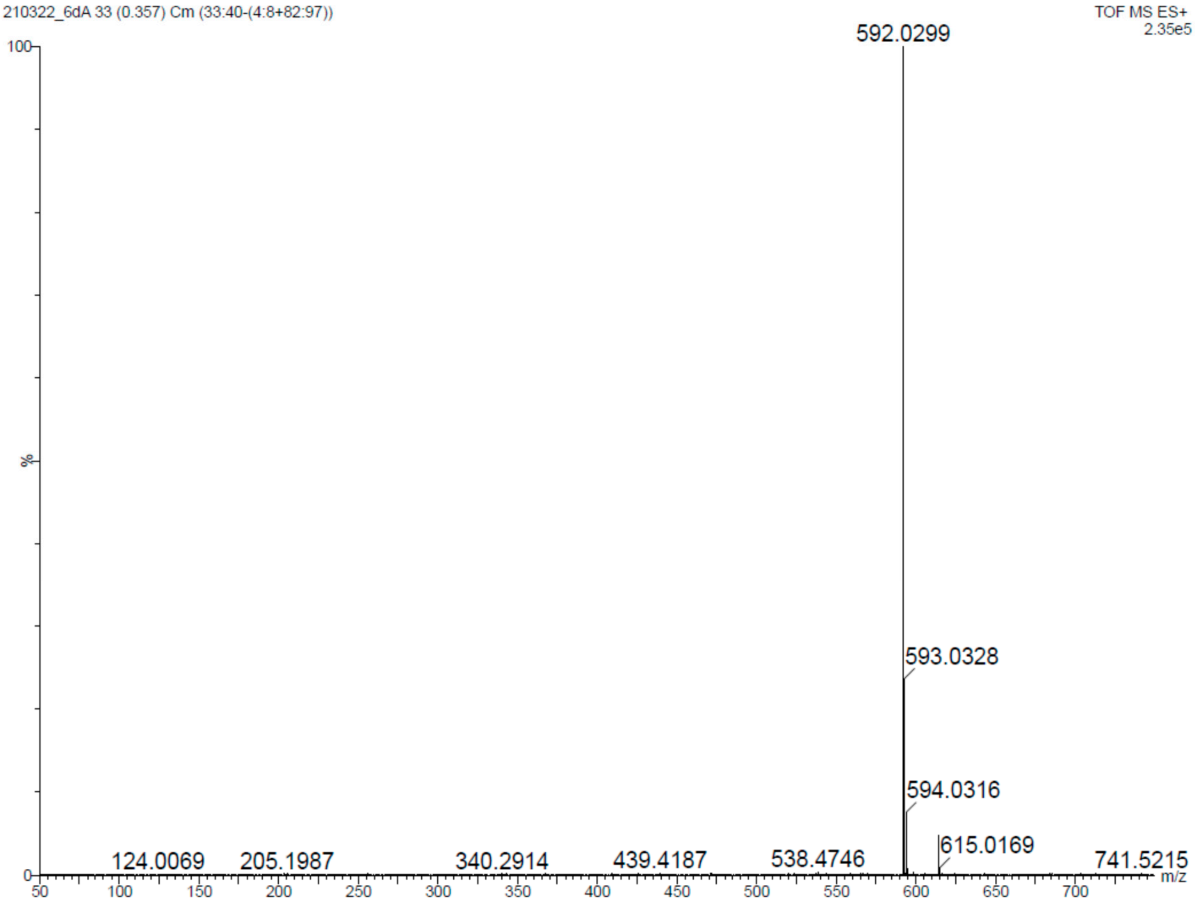
210322_6dA 33 (0.357) Cm (33:40-(4:8+82:97))
TOF MS ES+
2.35e5
592.0299
593.0328
594.0316
615.0169
124.0069
205.1987
340.2914
439.4187
538.4746
741.5215
100
%
0
50 100 150 200 250 300 350 400 450 500 550 600 650 700
m/z

Compound 6e

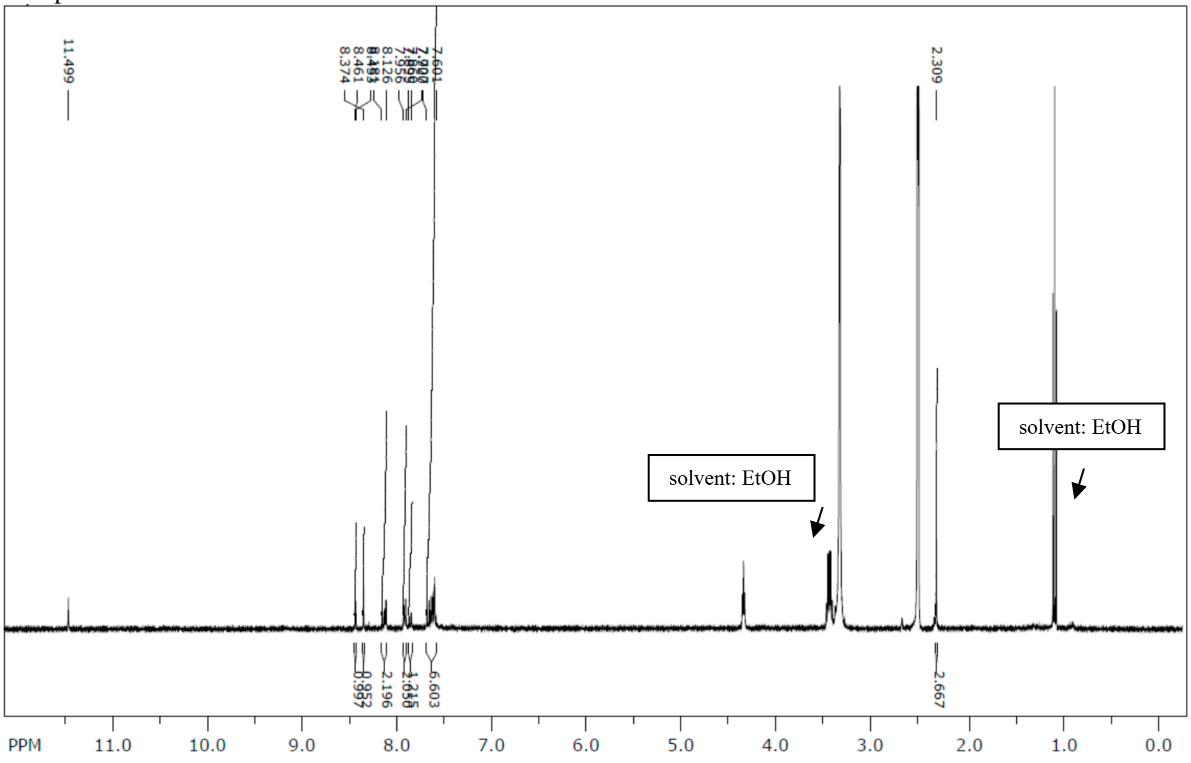

11.499
8.374
8.461
8.126
7.956
7.601
2.309
solvent: EtOH
solvent: EtOH
2.196
6.603
2.667
PPM
11.0
10.0
9.0
8.0
7.0
6.0
5.0
4.0
3.0
2.0
1.0
0.0

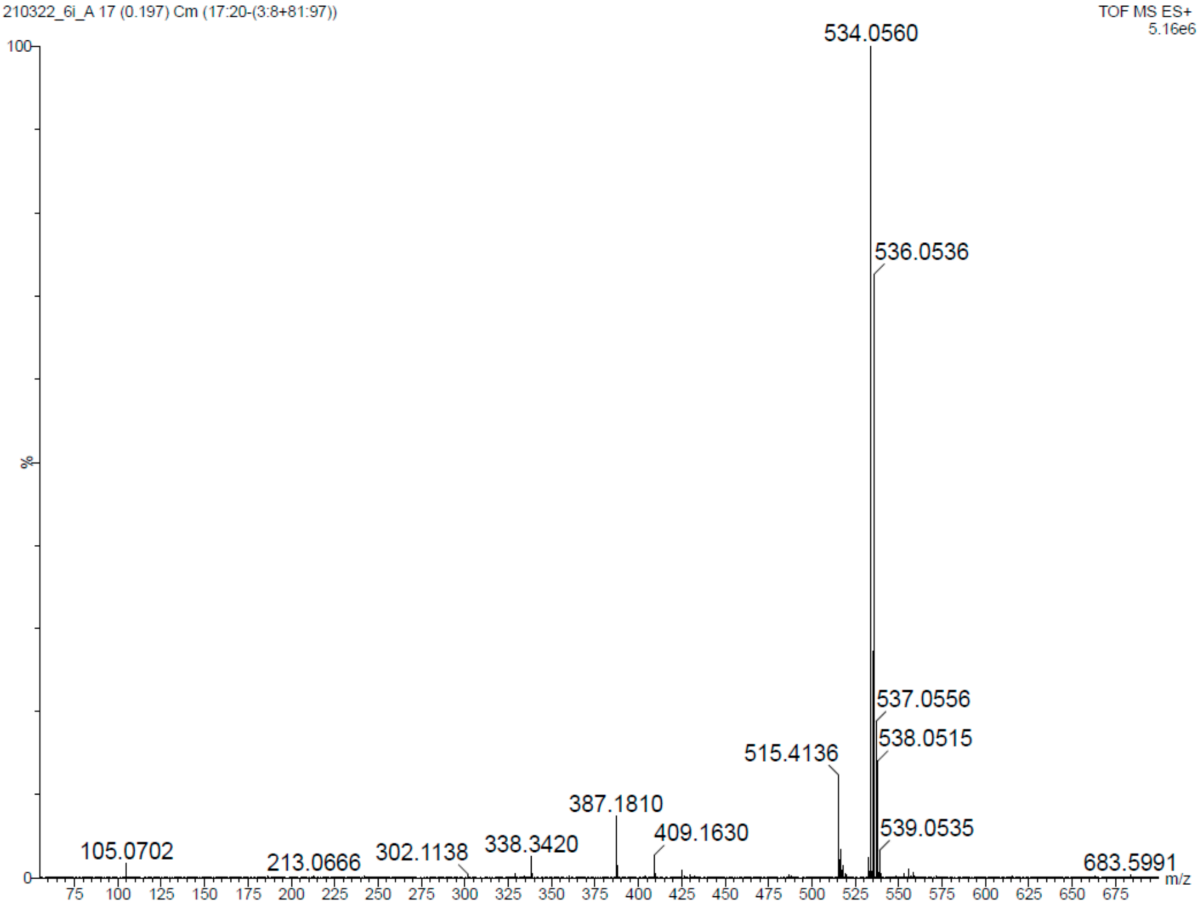

210322_6i_A 17 (0.197) Cm (17:20-(3:8+81:97))
TOF MS ES+
5.16e6
534.0560
536.0536
537.0556
538.0515
539.0535
515.4136
387.1810
409.1630
338.3420
302.1138
213.0666
105.0702
683.5991
100
%
0
m/z

Compound 6f

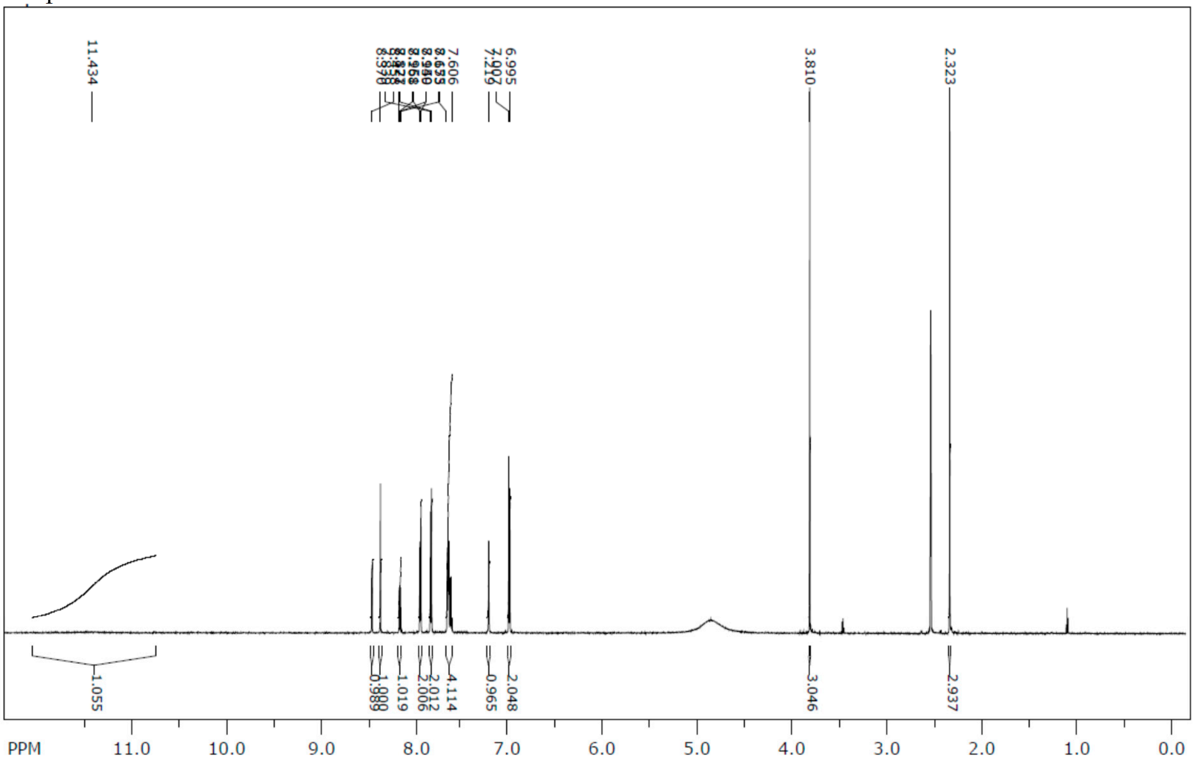

11.434
7.606
7.219
6.995
3.810
2.323
1.055
1.019
2.006
2.012
4.114
0.965
2.048
3.046
2.937
PPM
11.0
10.0
9.0
8.0
7.0
6.0
5.0
4.0
3.0
2.0
1.0
0.0

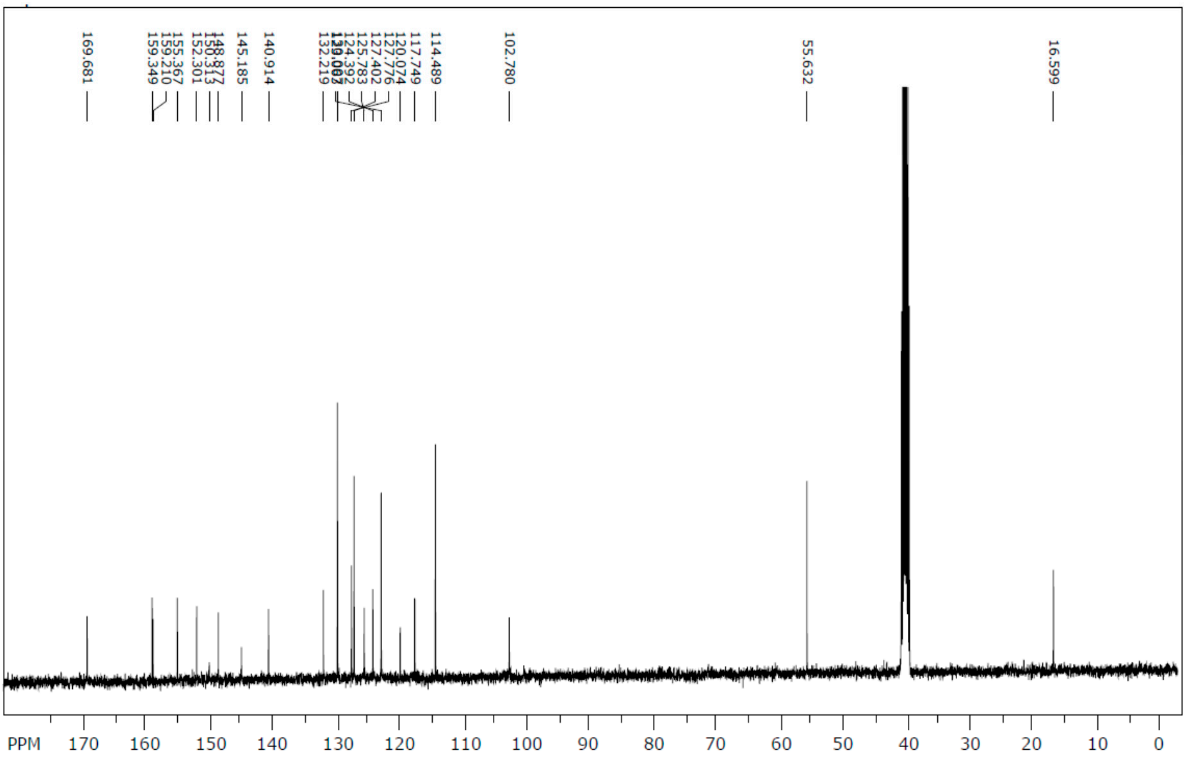

169.681
159.349
159.210
155.367
152.301
150.313
148.877
145.185
140.914
132.219
127.783
127.402
127.776
120.074
117.749
114.489
102.780
55.632
16.599
PPM
170
160
150
140
130
120
110
100
90
80
70
60
50
40
30
20
10
0

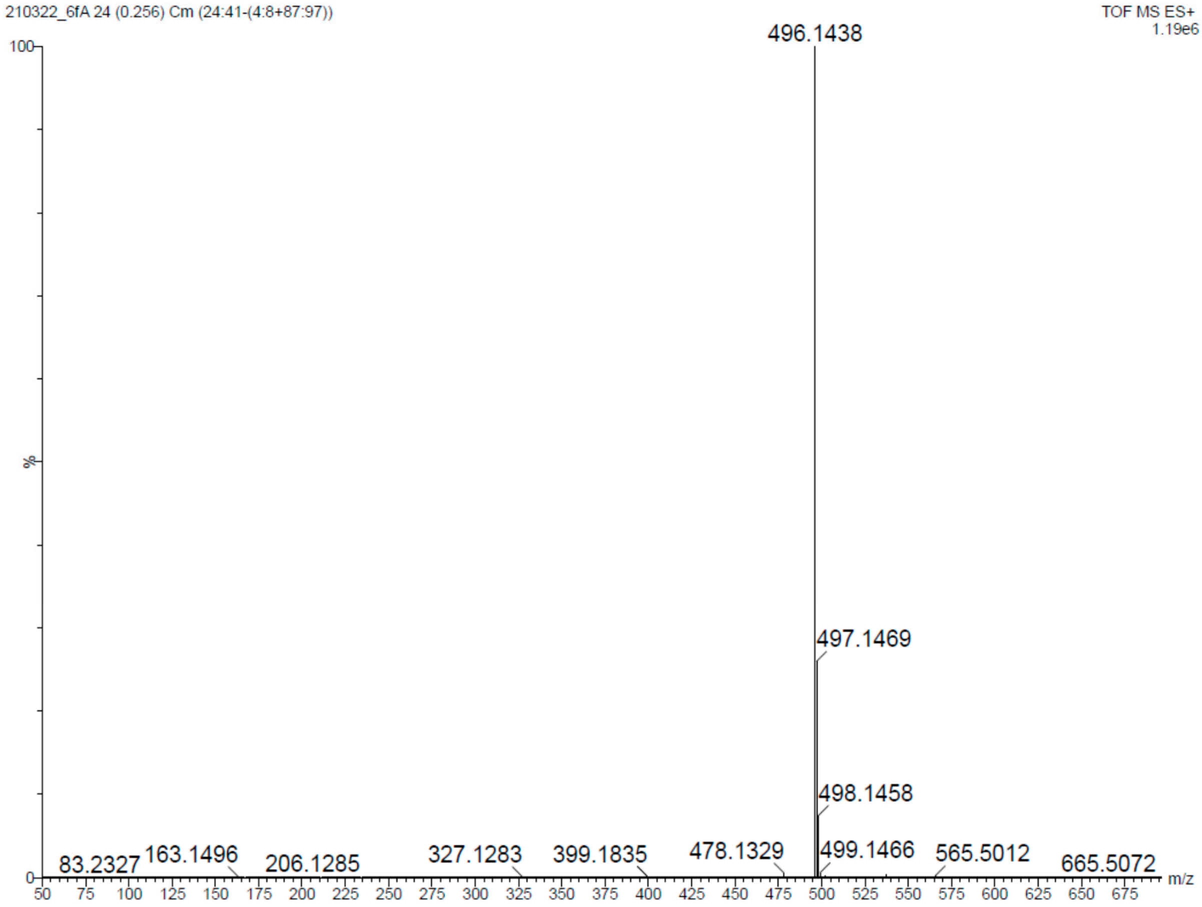
210322_6fA 24 (0.256) Cm (24:41-(4:8+87:97))
TOF MS ES+
1.19e6
496.1438
497.1469
498.1458
499.1466
83.2327
163.1496
206.1285
327.1283
399.1835
478.1329
565.5012
665.5072
%
m/z

Compound 6g

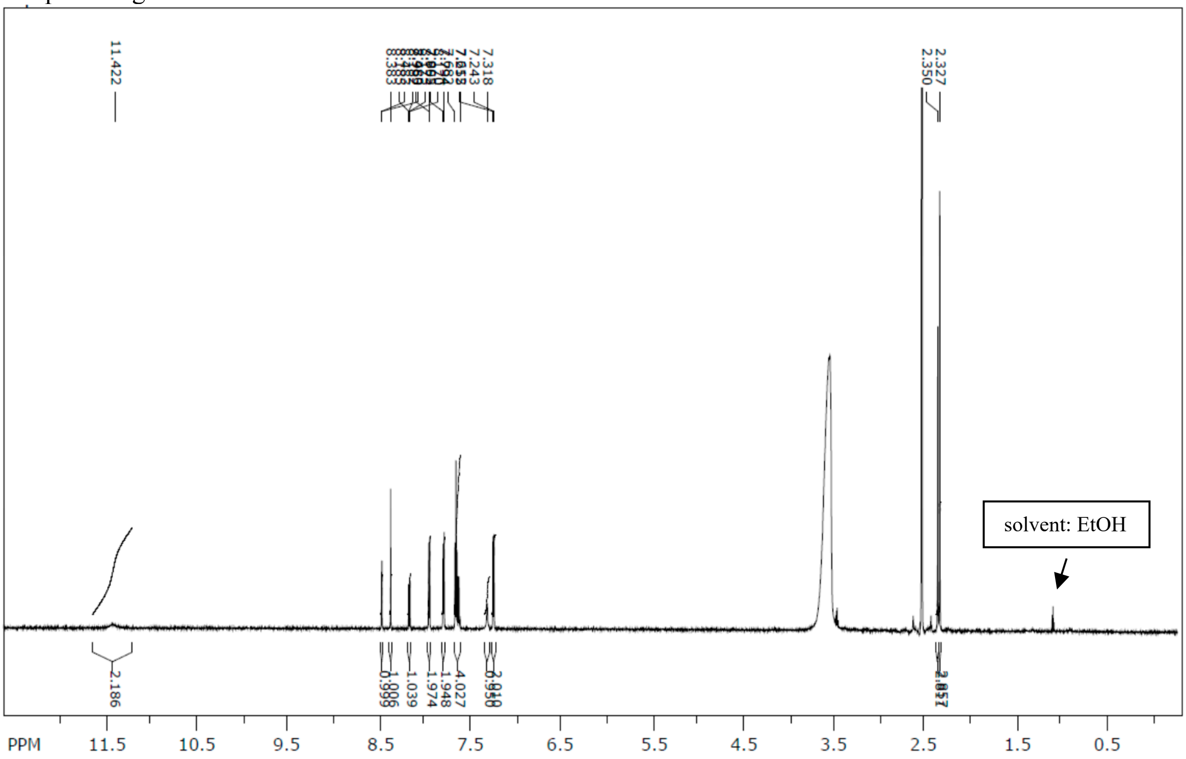
11.422
7.318
7.243
2.350
2.327
solvent: EtOH
2.186
1.039
1.974
1.948
4.027
PPM

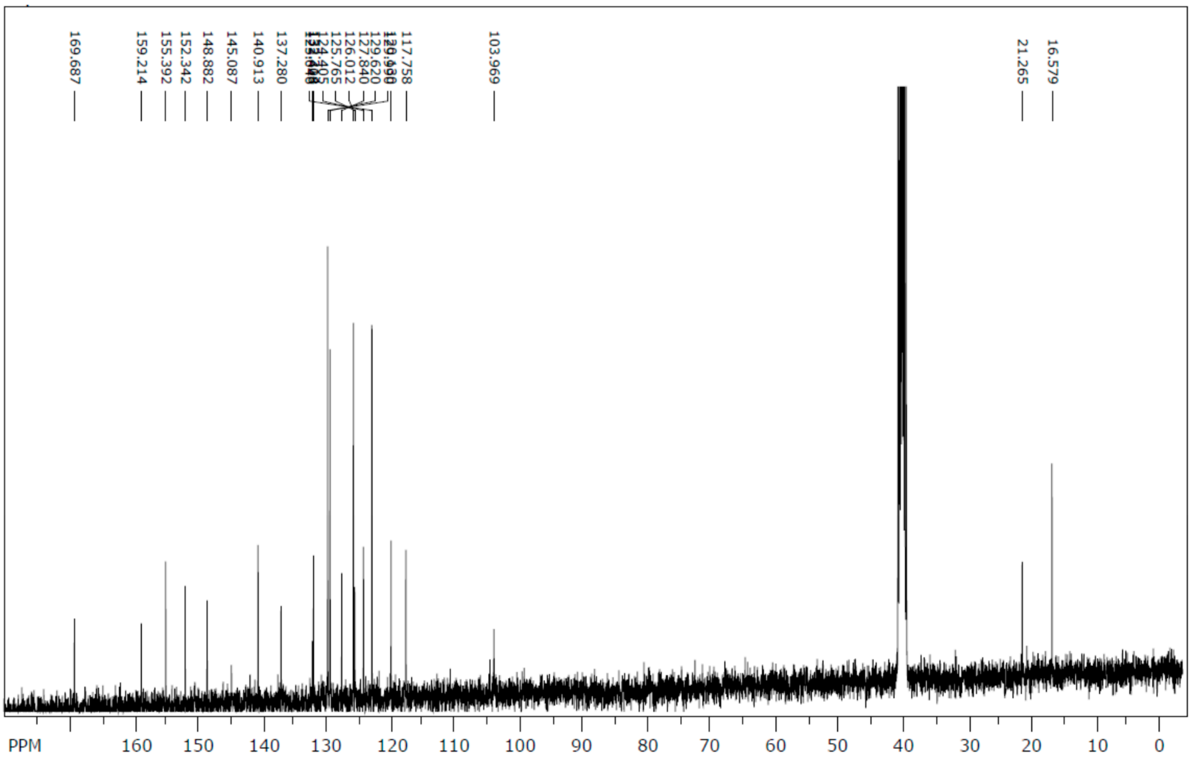
169.687
159.214
155.392
152.342
148.882
145.087
140.913
137.280
125.765
126.012
127.840
129.620
117.758
103.969
21.265
16.579
PPM

DEPT-135

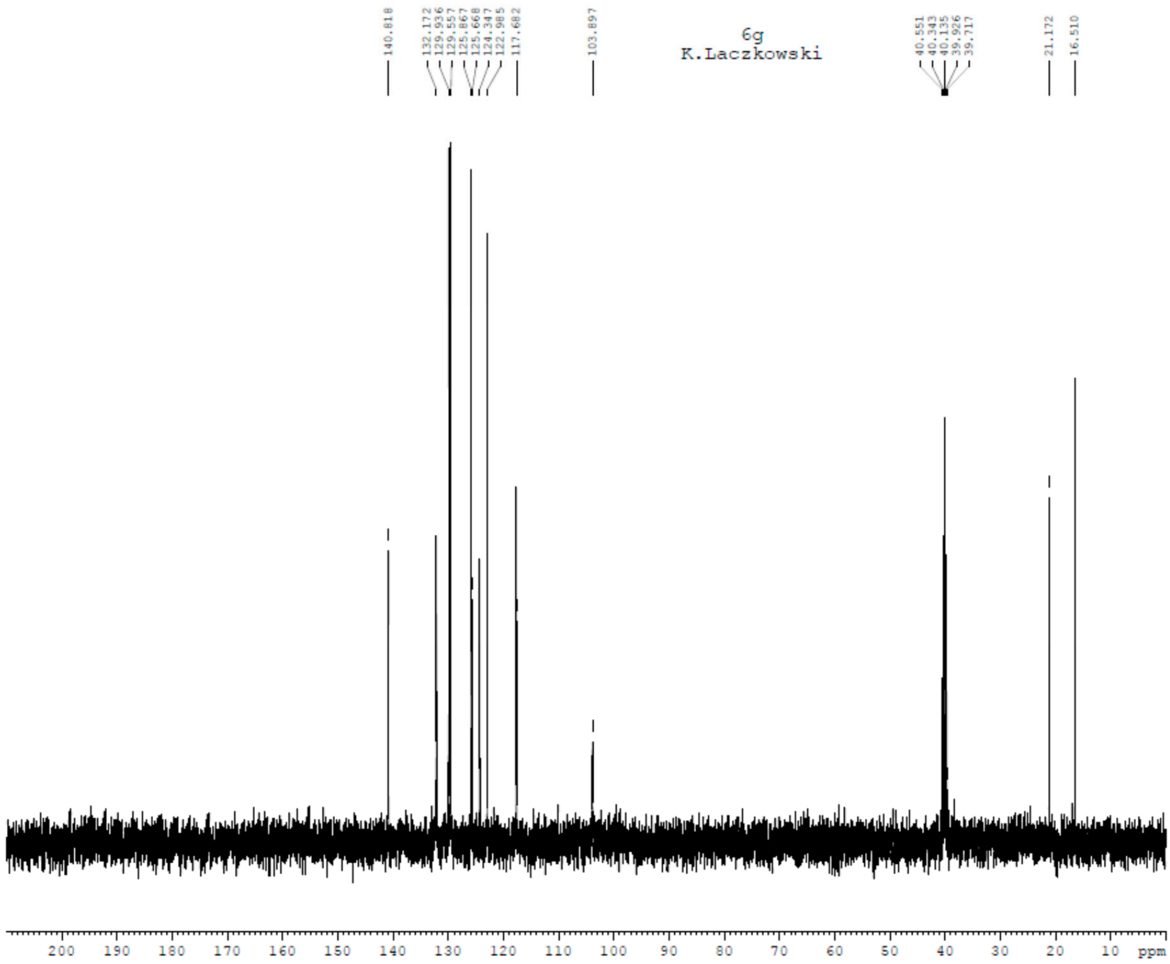
140.818
132.172
129.936
129.557
125.867
125.668
124.347
122.985
117.682
103.897
6g
K.Laczkowski
40.551
40.343
40.135
39.926
39.717
21.172
16.510
ppm

COSY

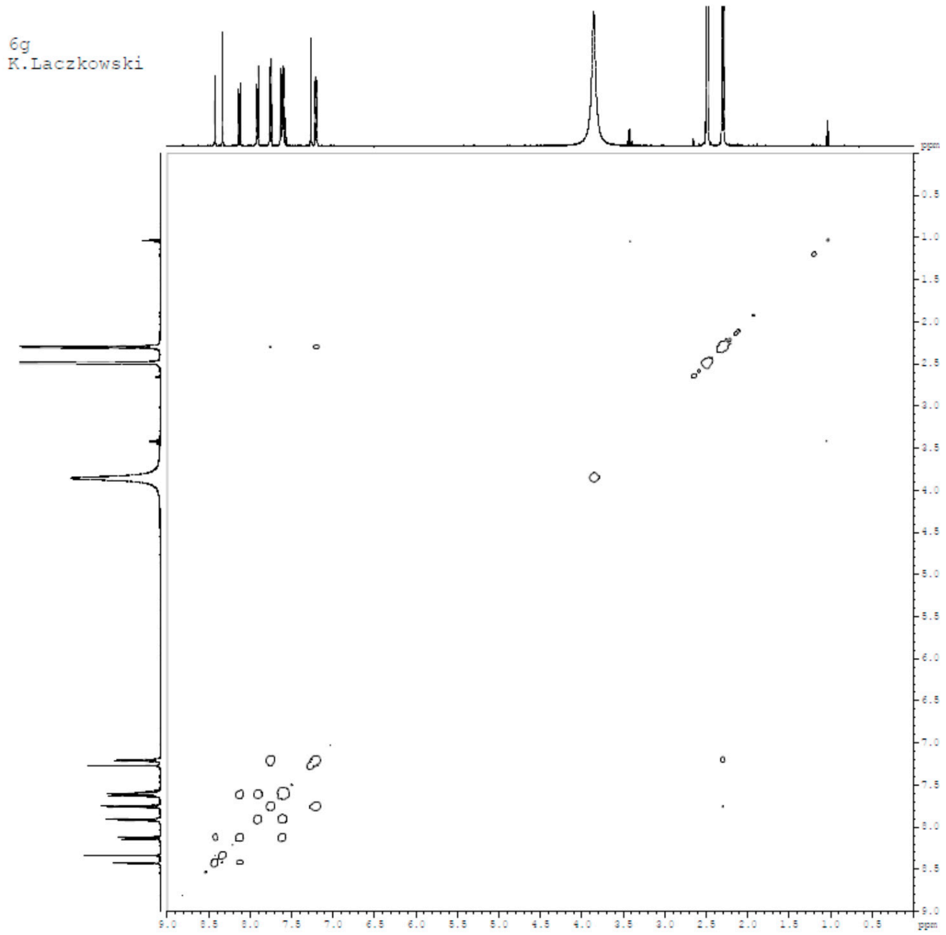

HMBC

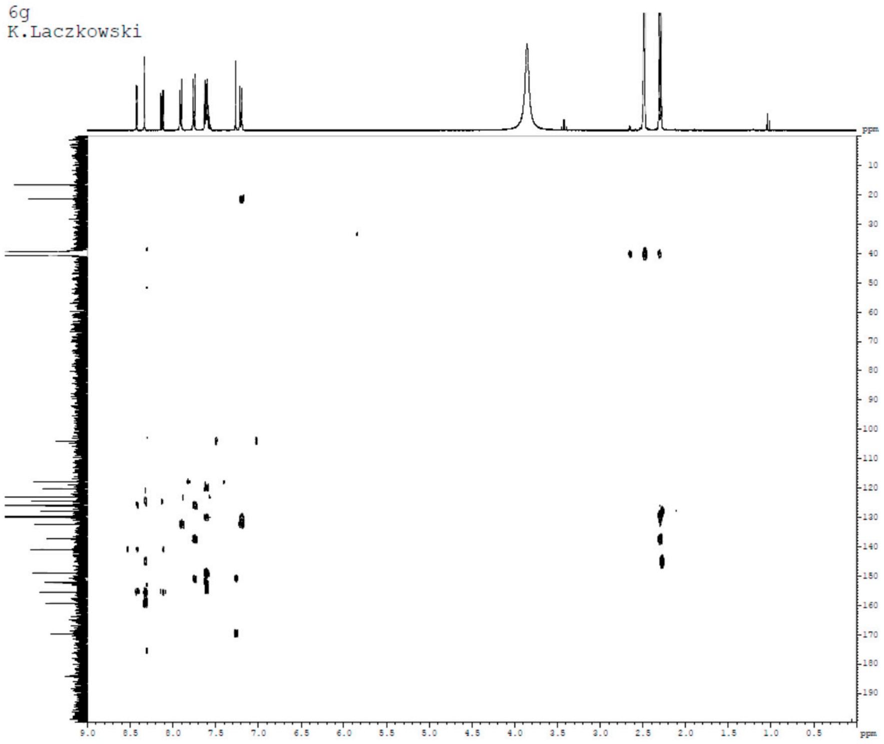

HSQC

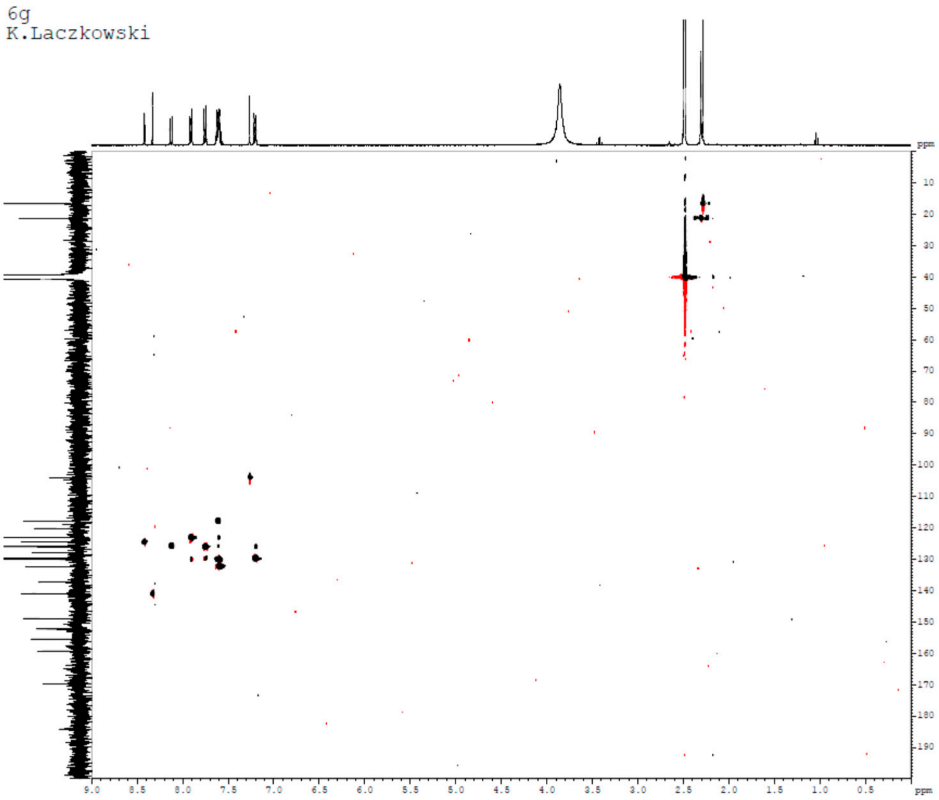

NOESY

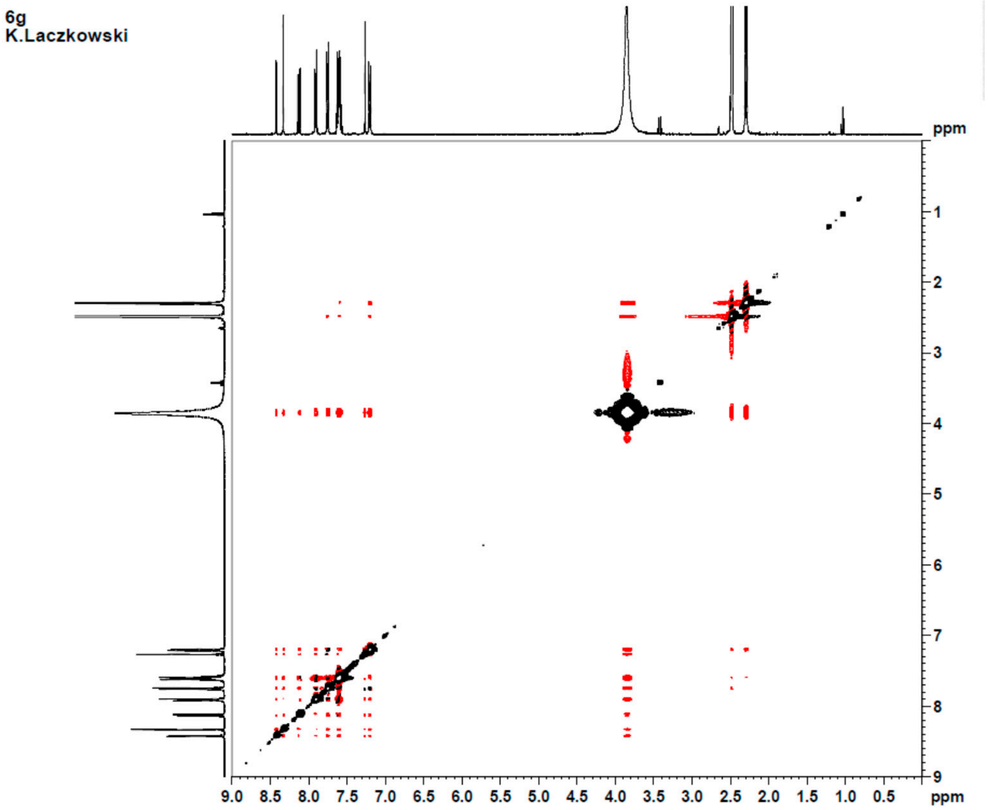

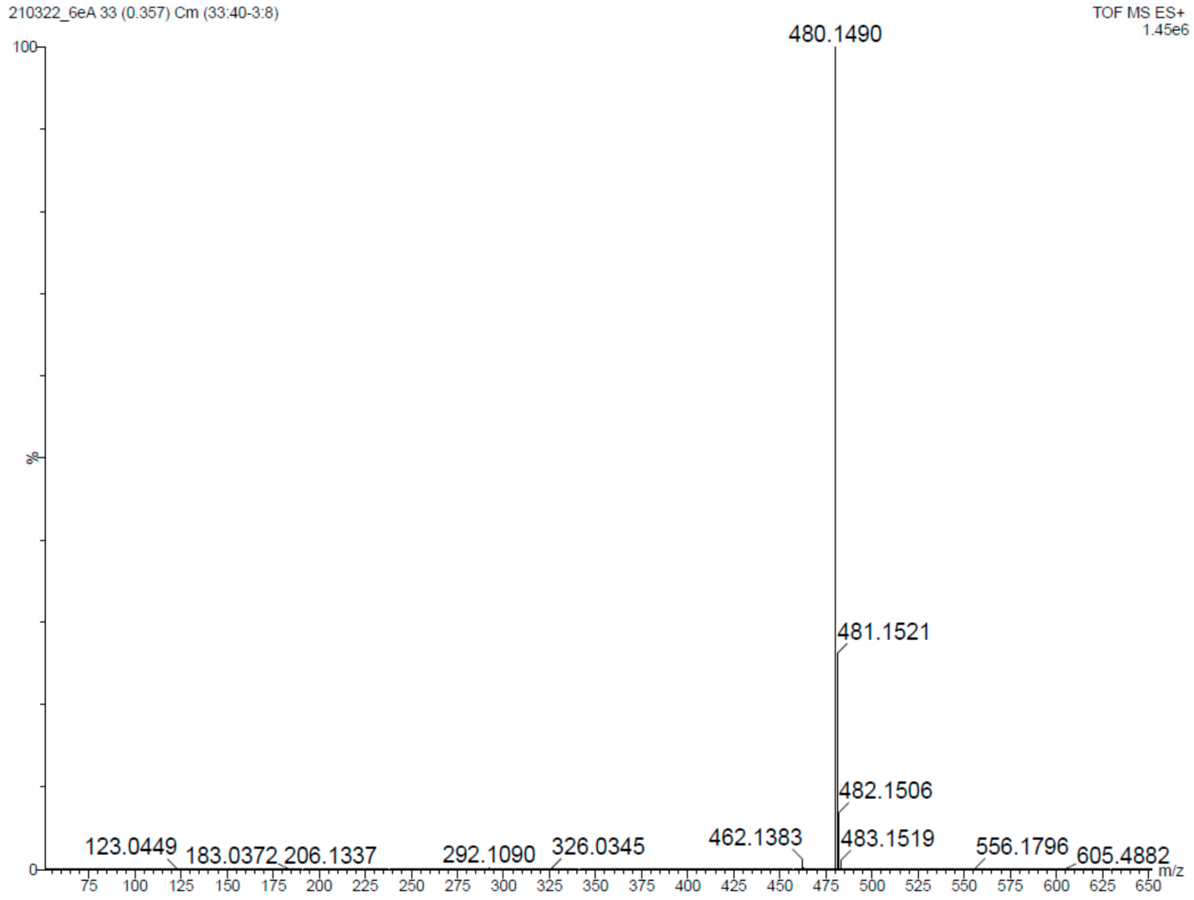
210322_6eA 33 (0.357) Cm (33:40-3:8)
TOF MS ES+
1.45e6
480.1490
481.1521
482.1506
483.1519
462.1383
123.0449
183.0372
206.1337
292.1090
326.0345
556.1796
605.4882
%
m/z

Compound 6h

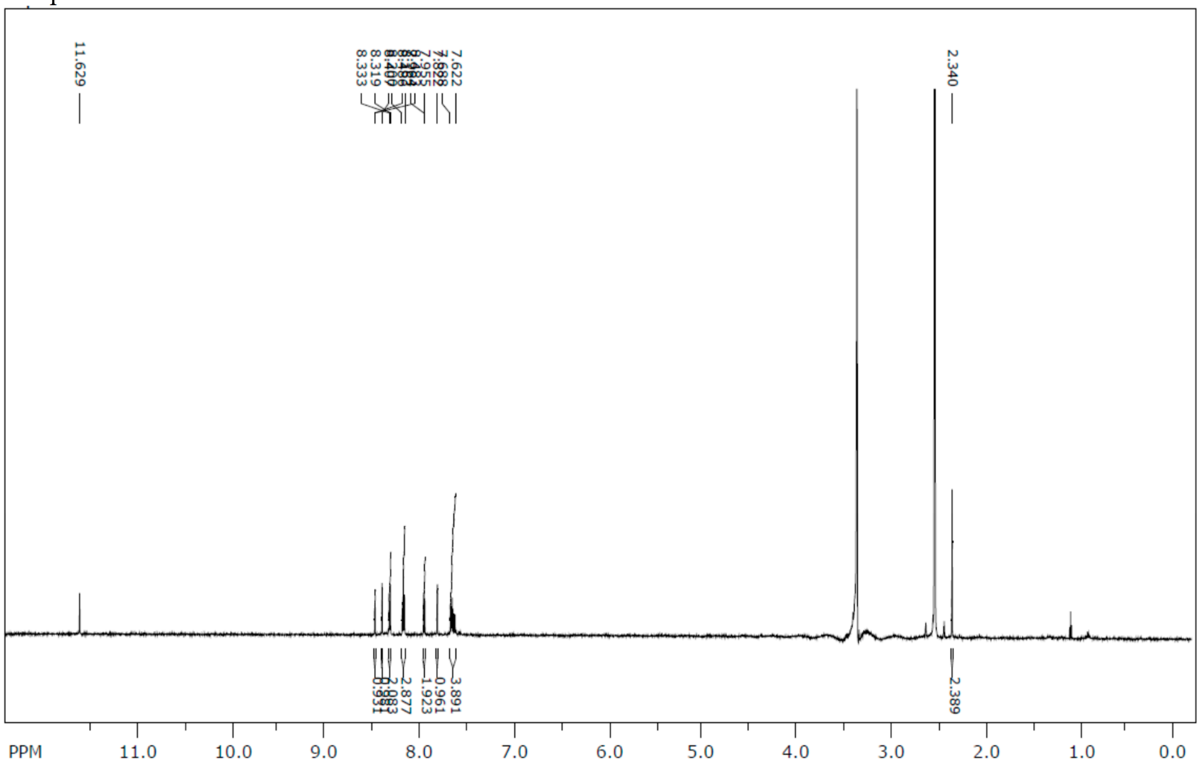
11.629
8.333
8.319
7.622
2.340
2.877
1.923
0.961
3.891
2.389
PPM

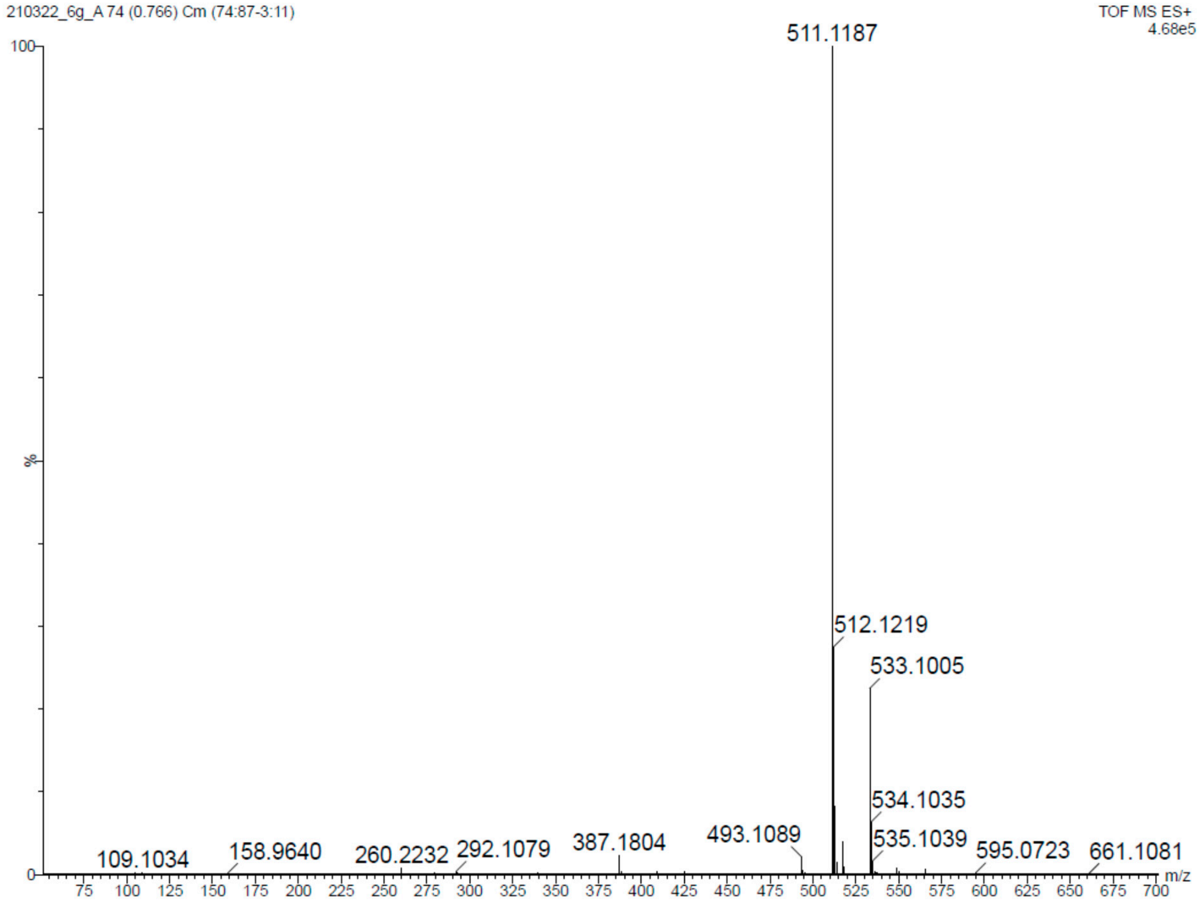

210322_6g_A 74 (0.766) Cm (74:87-3:11)
TOF MS ES+
4.68e5
511.1187
512.1219
533.1005
534.1035
535.1039
493.1089
387.1804
292.1079
260.2232
158.9640
109.1034
595.0723
661.1081
%
m/z

Compound 6i

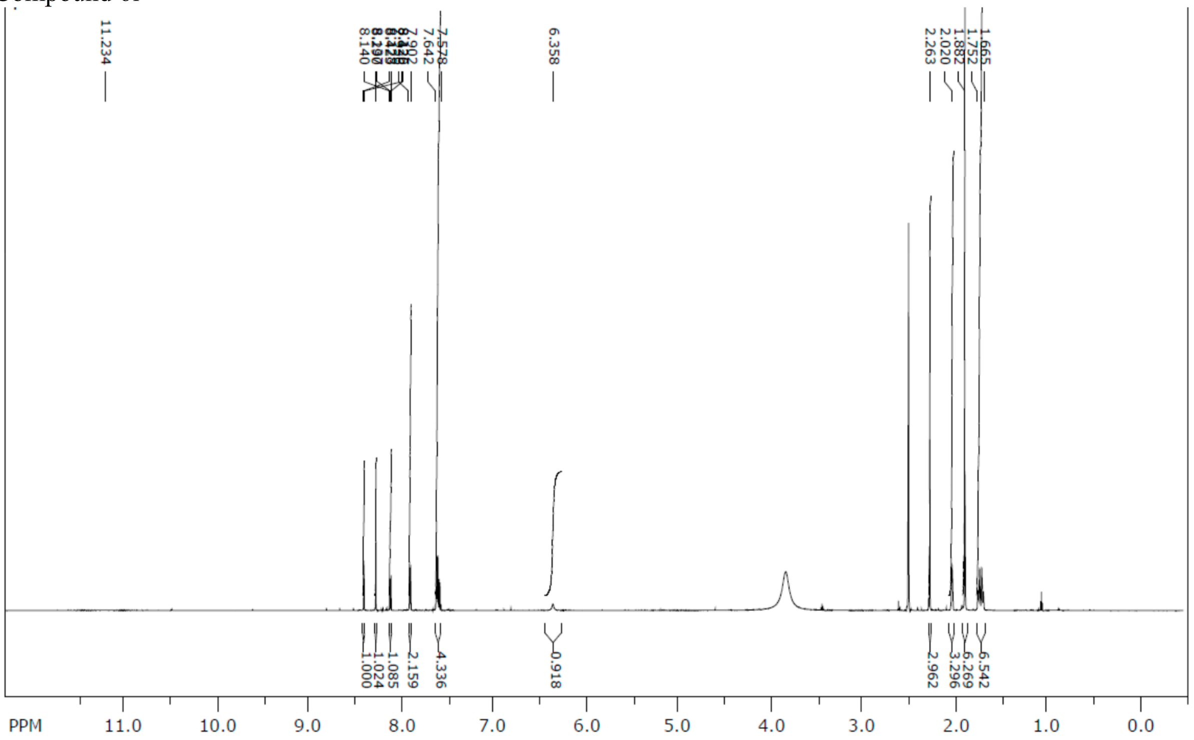

11.234
6.358
PPM
11.0
10.0
9.0
8.0
7.0
6.0
5.0
4.0
3.0
2.0
1.0
0.0

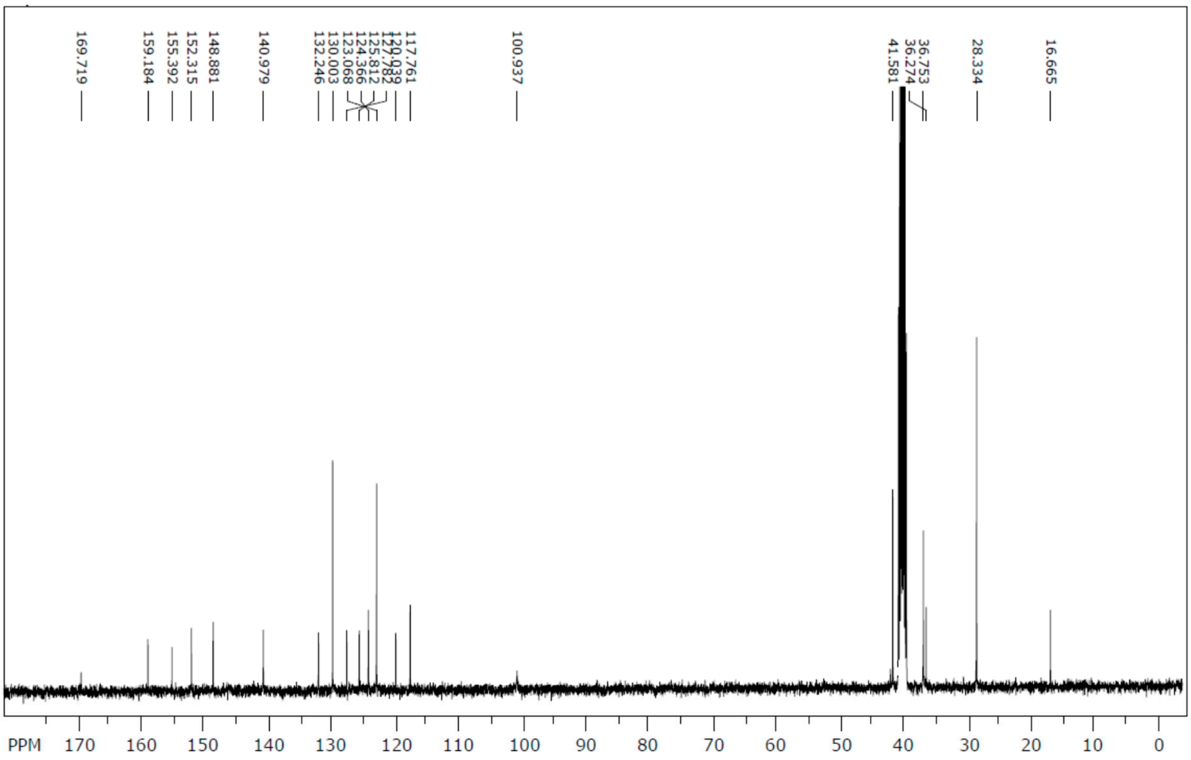

169.719
159.184
155.392
152.315
148.881
140.979
132.246
130.003
127.782
125.812
124.366
123.068
117.761
100.937
41.581
36.753
36.274
28.334
16.665
PPM 170 160 150 140 130 120 110 100 90 80 70 60 50 40 30 20 10 0

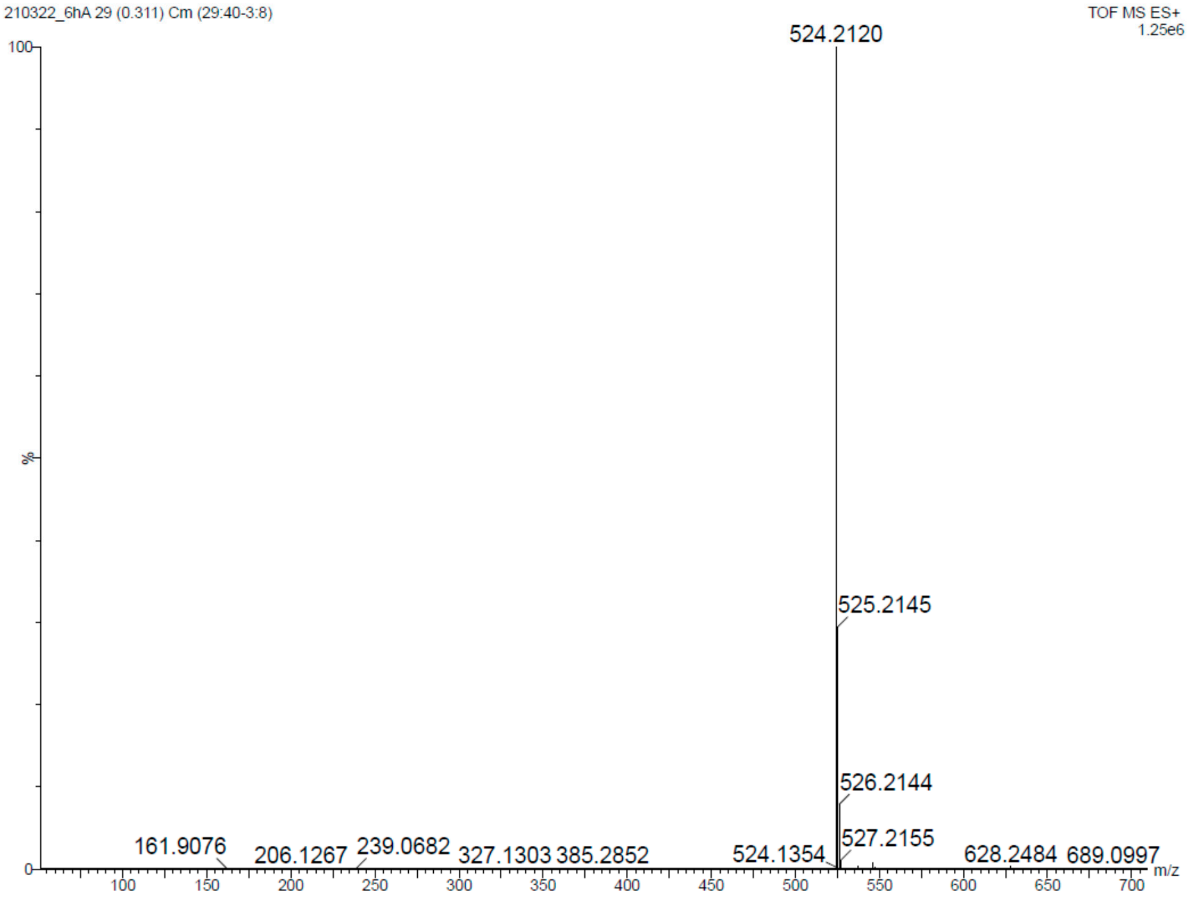

210322_6hA 29 (0.311) Cm (29:40-3:8)
TOF MS ES+
1.25e6
524.2120
525.2145
526.2144
527.2155
161.9076
206.1267
239.0682
327.1303
385.2852
524.1354
628.2484
689.0997
%
m/z
